# Harnessing *de novo* transcriptome sequencing to identify and characterize genes regulating carbohydrate biosynthesis pathways in *Salvia guaranitica* L.

**DOI:** 10.3389/fpls.2024.1467432

**Published:** 2024-09-26

**Authors:** Zahid Khorshid Abbas, Arwa Abdulkreem Al-Huqail, Aesha H. Abdel Kawy, Rabab A. Abdulhai, Doha A. Albalawi, Manal Abdullah AlShaqhaa, Moodi Saham Alsubeie, Doaa Bahaa Eldin Darwish, Ahmed Ali Abdelhameed, Fathia A. Soudy, Rania M. Makki, Maha Aljabri, Nadiah Al-Sulami, Mohammed Ali, Muhammad Zayed

**Affiliations:** ^1^ Department of Biology, Faculty of Sciences, University of Tabuk, Tabuk, Saudi Arabia; ^2^ Department of Biology, College of Science, Princess Nourah bint Abdulrahman University, Riyadh, Saudi Arabia; ^3^ Plant Ecophysiology Unit, Plant Ecology and Range Management Department, Desert Research Center, Cairo, Egypt; ^4^ Botany Department, Faculty of Women, Ain Shams University, Cairo, Egypt; ^5^ Biodiversity Genomics Unit, Faculty of Science, University of Tabuk, Tabuk, Saudi Arabia; ^6^ Department of Biology, College of Science, King Khalid University, Abha, Saudi Arabia; ^7^ Biology Department, College of Science, Imam Mohammad Ibn Saud Islamic University (IMSIU), Riyadh, Saudi Arabia; ^8^ Agricultural Botany Department (Genetics), Faculty of Agriculture, Al-Azhar University, Assuit, Egypt; ^9^ Genetics and Genetic Engineering Department, Faculty of Agriculture, Benha University, Moshtohor, Egypt; ^10^ Department of Biological Sciences, Faculty of Science, King Abdulaziz University (KAU), Jeddah, Saudi Arabia; ^11^ Department of Biology, Faculty of Science, Umm Al-Qura University, Makkah, Saudi Arabia; ^12^ Maryout Research Station, Genetic Resources Department, Desert Research Center, Cairo, Egypt; ^13^ Department of Botany and Microbiology, Faculty of Science, Menoufia University, Shebin El-Kom, Egypt

**Keywords:** *Salvia guaranitica*, transcriptome, glycolysis, gluconeogenesis, starch, sucrose, transgenic *Arabidopsis thaliana*, functional characterization

## Abstract

**Introduction:**

Carbohydrate compounds serve multifaceted roles, from energy sources to stress protectants, found across diverse organisms including bacteria, fungi, and plants. Despite this broad importance, the molecular genetic framework underlying carbohydrate biosynthesis pathways, such as starch, sucrose, and glycolysis/gluconeogenesis in *Salvia guaranitica*, remains largely unexplored.

**Methods:**

In this study, the Illumina-HiSeq 2500 platform was used to sequence the transcripts of *S. guaranitica* leaves, generating approximately 8.2 Gb of raw data. After filtering and removing adapter sequences, 38 million reads comprising 210 million high-quality nucleotide bases were obtained. De novo assembly resulted in 75,100 unigenes, which were annotated to establish a comprehensive database for investigating starch, sucrose, and glycolysis biosynthesis. Functional analyses of glucose-6-phosphate isomerase (*SgGPI*), trehalose-6-phosphate synthase/phosphatase (*SgT6PS*), and sucrose synthase (*SgSUS*) were performed using transgenic *Arabidopsis thaliana*.

**Results:**

Among the unigenes, 410 were identified as putatively involved in these metabolic pathways, including 175 related to glycolysis/gluconeogenesis and 235 to starch and sucrose biosynthesis. Overexpression of *SgGPI*, *SgT6PS*, and *SgSUS* in transgenic A. thaliana enhanced leaf area, accelerated flower formation, and promoted overall growth compared to wild-type plants.

**Discussion:**

These findings lay a foundation for understanding the roles of starch, sucrose, and glycolysis biosynthesis genes in *S. guaranitica*, offering insights into future metabolic engineering strategies for enhancing the production of valuable carbohydrate compounds in *S. guaranitica* or other plants.

## 1 Introduction

*Salvia guaranitica* belongs to the Lamiaceae family. It is a valuable medicinal and fragrant plant that is native to South America and is widely cultivated in moderate and tropical climates across the globe, particularly in Uruguay, Paraguay, Brazil, Egypt, China, West Asia, and East Asia (Abd El-Wahab et al., 2015; Ali et al., 2018; Santa Cruz et al., 2021; Ali, 2023). The genus *Salvia* comprises numerous species that are marketed in traditional medicine for their healing properties, including *S. officinalis, S. aegyptiaca, S. japonica, S. acerifolia, S. santolinifolia, S. acuminata, S. hydrangea, S. aethiopis, S. tomentosa, S. africana, S. tuxtlensis, S. africana, S. miltiorrhiza, S. arrabidae, S. chloroleuca, S. amplifrons, S. nipponica, S. algeriensis, S. fruticosa, S. aureus, S. amplifrons, S. przewalskii, S. argentea, S. epidermindis, S. isensis, S. arabica, S. arizonica, S.aethiopis, S. aequidens, and S. arenaria*, as listed in the Plant List database of the World Flora Online (WFO) http://www.theplantlist.org/1.1/browse/A/Lamiaceae/Salvia/. Most sage species possess a significant concentration of essential oils, notably monoterpenes and sesquiterpenes (Ali et al., 2017, 2018, 2022c, 2022a, 2022b; El-ramah et al., 2022; Khater, 2022).

In plants, carbohydrates such as glucose, sucrose, trehalose, and starch are mono-, di-, and polysaccharides that are created during photosynthesis (Trouvelot et al., 2014). These carbohydrates and other essential chemicals are produced by numerous metabolic processes; including glycolysis/gluconeogenesis (KEGG: map00010), citrate cycle (KEGG: map00020), pentose phosphate pathway (KEGG: map00030), pentose and glucuronate interconversions (KEGG: map00040), fructose and mannose metabolism (KEGG: map00051), ascorbate and aldarate metabolism (KEGG: map00053), starch and sucrose metabolism (KEGG: map00500), amino sugar and nucleotide sugar metabolism (KEGG: map00520), pyruvate metabolism (KEGG: map00620), glyoxylate and dicarboxylate metabolism (KEGG: map00630), propanoate metabolism (KEGG: map 00640), butanoate metabolism (KEGG: map00650), C5-branched dibasic acid metabolism (KEGG: map 00660) and inositol phosphate metabolism (KEGG: map00562) (accessed on 5 Mar 2024) (Kanehisa, 2000, 2019; Kanehisa et al., 2023). Carbohydrate compounds are crucial for various biological processes in plants, including photosynthesis, plant development, signaling, growth, defense mechanisms, biochemical processes within plant cells, regulation of defense genes, plant immunity, symbiotic interactions in legume plants, and coordination of metabolism in response to biotic and abiotic stresses caused by environmental changes. Additionally, carbohydrates also serve as antioxidants and help regulate the generation of reactive oxygen species (ROS) (Koch, 1996, 2004; Sheen et al., 1999; Rolland et al., 2002, 2006; Smeekens et al., 2010; Walters et al., 2013; Keunen et al., 2013; Delaunois et al., 2014; Ali et al., 2022a, 2022b, 2022c). Consequently, breeders and biotechnology experts are primarily focused on augmenting the levels of diverse carbohydrate molecules in plants and crops (Kanehisa et al., 2023).

Currently, RNA-Sequencing (RNA-Seq), a Next-Generation Sequencing (NGS) technology, has emerged as a powerful tool for gene identification. It serves as a significant approach for discovering new genes and elucidating their associations with specific metabolic pathways (Li et al., 2018, 2023; Badawi et al., 2019; Safavi-Rizi et al., 2020; Zayed and Badawi, 2020; Mehmood et al., 2021; Bairakdar et al., 2023; Kanehisa et al., 2023). For example, the starch metabolism in barley grain (Collins et al., 2021), starch accumulation and biosynthesis in sorghum seeds (Ke et al., 2022; Xiao et al., 2022), starch metabolism in *Triticum aestivum*, and *Fagopyrum tataricum* (Gu et al., 2021; Huang et al., 2022), starch metabolism in *Castanea henryi* seeds (Liu et al., 2020), regulator of starch synthesis in *Oryza sativa* (Liu et al., 2022), starch biosynthesis in *Zea mays* (Zhang et al., 2019). Trehalose metabolism and biosynthesis in *T. eastivum*, *Manihot esculenta*, *Medicago truncatula*, *Zea mays* (Macovei et al., 2019; Luo et al., 2021; Lyra et al., 2021; Sukko et al., 2023), and sucrose metabolism in *Z. mays*, *Ipomoea batatas*, *Vitis vinifera*, and *Arachis hypogaea* (Zhu et al., 2017; Li et al., 2021; Jiang et al., 2023).

Recently, there have been several research on plant genes implicated in glycolysis/gluconeogenesis, starch and sucrose production and regulation. Furthermore, glycolysis/gluconeogenesis, starch, and sucrose regulatory genes from several plant species were discovered, cloned, and characterized, and then employed for engineering the metabolism of many plant species (Huang et al., 2022). For examples, cloning genes encoding the starch biosynthetic enzymes from *O. sativa, F. tataricum* and *Z. mays* (Jeon et al., 2010; Huang et al., 2021, 2022), sucrose-phosphate synthase (SPS), and sucrose synthase (SuSy) from *Z. mays*, *Solanum lycopersicum*, and *Saccharum* spp. hybrids (Nguyen‐Quoc and Foyer, 2001; Chandra et al., 2015; Xiao et al., 2024), and trehalose-6-phosphate synthase besides trehalose-6-phosphate phosphatase from *A. thaliana, Rosa hybrida, Citrus sinensis* (Singh et al., 2011; Liu and Zhou, 2022; Fan et al., 2023). Given that the genes for glycolysis/gluconeogenesis, starch and sucrose biosynthesis and regulation are unknown in *S. guaranitica*, this study focuses on identifying the candidate genes that are associated with glycolysis/gluconeogenesis, starch, and sucrose biosynthesis from *S. guaranitica*. Therefore, the identification and functional characterization of three enzymes-encoding genes from *S. guaranitica* which are glucose-6-phosphate isomerase (*SgGPI*), trehalose 6-phosphate synthase/phosphatase (*SgT6PS*) and sucrose synthase (*SgSUS*) is the focal point to elucidate glycolysis/gluconeogenesis, starch, and sucrose biosynthesis from *S. guaranitica*. Also, our work resolves their biological roles when transformed into the model plant, *A. thaliana*. These results illustrate the functioning of the regulatory network governing glycolysis/gluconeogenesis, starch, and sucrose metabolism in *S. guaranitica*. Moreover, the results provide valuable genetic resources for enhancing plant traits.

## 2 Materials and methods

### 2.1 Plant sampling, RNA library preparation, and sequencing

To study the transcriptome profiles, three biological replicates were collected using three separate *S. guaranitica* plants. Each replicate included a pooled sample of both young and mature leaves. The plants utilized in this study were two years old. Moreover, three biological replicates were gathered from mature leaves, tender leaves, flowering parts, flower buds, stems, and roots for the qRT-PCR assays. The samples were swiftly stored in liquid nitrogen and thereafter held onto -80°C till needed (Mehmood et al., 2021). TRIzol™ Reagent (Invitrogen, CA, US) was employed to obtain the total RNA from different samples as per the guidelines provided by the manufacturer. After treating the extracted RNA from various samples with DnaseI (Takara, China), its overall quality was assessed by subjecting it to electrophoresis on a 1.25% agarose-formaldehyde gel, followed by visualization with ethidium bromide. Additionally, the NanoDrop™ 2000/2000c Spectrophotometers (MA, USA) was utilized to calculate the RNA quality and concentration from different samples. Ten µg of RNA was used for cDNA synthesis via the reverse transcription kit (M-MLV, China) (Huang et al., 2012; Rastogi et al., 2014; Zhu et al., 2019; Santa Cruz et al., 2021). High-quality RNAs extracted from different samples were utilized to construct cDNA libraries (Ali et al., 2017, 2018). Sequencing was achieved on the high-quality libraries using an Illumina HiSeq 2500 platform, generating paired-end reads. Clean reads were acquired by filtering out adapters, poly-N sequences, and low-quality reads. Subsequently, the assembly was performed by the Trinity platform https://github.com/trinityrnaseq/trinityrnaseq/wiki with the parameters “min_kmer_cov set to 2”. Subsequently, the values of Q20, Q30, GC content, and sequence duplication level were estimated (Yang et al., 2017).

### 2.2 *In silico* differential gene expression and protein domain analysis

To examine the putative transcription levels of *SgGPI, SgT6PS*, and *SgSUS* across various tissues, the *A. thaliana* eFPbrowsers (http://bar.utoronto.ca/efp/cgi-bin/efpWeb.cgi) was employed. Moreover, the predicted subcellular localizations of their orthologs from *A. thaliana* were retrieved. Subsequently, the image envisioned their cellular localizations was built, and then the putative domains were estimated through the InterPro database (https://www.ebi.ac.uk/interpro/) (El-ramah et al., 2022; Elsherbeny et al., 2022; Makhadmeh et al., 2022a, 2022b; Abdelhameed et al., 2024b).

### 2.3.Validation and relative expression analysis of glycolysis/gluconeogenesis, starch and sucrose metabolism genes

Towards examining the activity levels of glycolysis/gluconeogenesis, starch, and sucrose biosynthesis genes in S. guaranitica at different tissues, twenty candidate genes were chosen. The expression profiles for these selected genes were compared within various tissue samples to disclose their ‘transcriptional control’, offering insights into the epistatic relationship regarding mRNA copies, and the products and the end-products. The expression profiles of our chosen candidates: *SgGPI, SgT6PS, SgSUS, SgPFK9, SgALDH, SgALDO, SgPYK, SgFBP, SgACS, SgPCKA, SgGlGA, SgGlGC, SgBMY, SgGBE1, SgAGL, SgBGL, SgHK, SgPYG, SgUGDH* and *SgINV*, across different tissues were investigated.

### 2.4 Cloning of full-length starch, sucrose and glycolysis synthase cDNAs

The *SgGPI, SgT6PS*, and *SgSUS* full-length cDNAs were amplified using gene-specific primers designed from the Illuimina sequencing data of *S. guaranitica* leaves (**Supplementary Table S1**). The initial PCR was conducted with the short primers, KOD-Plus-DNA polymerase (Toyobo, Japan), and leaf cDNA using the program: 96°C for 4 min, 98°C for 12 s, 60°C for 4 s, 68°C for 2.5 min, and 34 cycles followed by 68°C for 15 min. Regarding the following PCR, the products from the initial PCR were used as templates with the long primers under the same PCR conditions. The products were then cleaned and then transferred to the Gateway entry vector (pDONR221), then subsequently sub-cloned into the destination vector (pB2GW7). The vectors were incorporated into *A. thaliana* flowers via *Agrobacterium tumefaciens* strain GV3101 by electroporation. The cloning steps were verified using Sanger sequencing (Rehman et al., 2018; Ali et al., 2022a, 2022c, 2022b).

### 2.5 Functional characterization of *SgGPI*, *SgT6PS* and *SgSUS* in transgenic *A. thaliana* leaves

The genes *SgGPI*, *SgT6PS* and *SgSUS* were chosen to be characterized and expressed in *A. thaliana* utilizing the *Agrobacterium*-mediated floral dip technique. The transformation was performed using *A. tumefaciens* GV3101 harboring pB2GW7-*SgGPI*, pB2GW7-*SgT6PS*, and pB2GW7-*SgSUS* plasmids driven by the 35S promoters. Using the procedure outlined by (Ali et al., 2018, 2022a, 2022c, 2022b; Ali, 2023). Briefly, the *A. thaliana* seeds were germinated in advance and the plants were made ready for transformation after two months. The secondary inflorescences were immersed in a solution containing *Agrobacterium* carrying the pB2GW7-vector, specifically targeting the gynoecium of the flower. The plants were cultivated until the siliques reached a brown and dried state. Subsequently, the seeds were collected, cultivated again, and subjected to BASTA treatment – a herbicide containing glufosinate-ammonium – to select the desired transgenic seedlings carrying the resistance gene against BASTA. Moreover, the presence of target genes in positive transgenic lines was verified through semi-quantitative RT-PCR (semi-qRT-PCR). The physiological and biochemical parameters of different transgenic lines were evaluated. A total of twelve 45-day-old plants, including putative transgenic and wild-type plants, were chosen for the purpose of harvesting mature leaves. These leaves were then subjected to semi-qRT-PCR for measuring the activity levels of the Salvia-derived genes (*SgGPI, SgT6PS* and *SgSUS*) into transgenic *A*. *thaliana* plants.

### 2.6 Determination of relevant physiological and biochemical indices

Soluble sugars, including sucrose, glucose, and fructose, were analyzed as described by (Sonnewald et al., 1991; Ortiz-Marchena et al., 2014). Briefly, the quantification involved extraction from both wild-type and genetically modified *A. thaliana* plant leaves using 80% ethanol in 10 mM HEPES-KOH (pH 7.7) at 80°C for 2 hours. The supernatant was utilized to measure glucose, fructose, and sucrose concentrations through the sequential addition of specific enzymes—5 units each of glucose-6-phosphate dehydrogenase and hexokinase, 2 units of glucose-6-phosphate isomerase, and 20 units of invertase—followed by the monitoring of NAD^+^ reduction at 340 nm absorbance at various intervals.

Each parameter was tested with three biological replicates. Furthermore, the quantities of chlorophyll a, b, and total (a+b), were measured following (El-Mahdy et al., 2024). Concisely, about 20-30 mg of fresh leaf samples were weighted from each treatment, then each sample was transferred to a centrifuge tube with 4 mL dimethylformamide (DMF) and maintained away from light to preserve chlorophyll integrity. The level of chlorophyll a, b, and total (a+b) in the extracts were accomplished following (El-Mahdy et al., 2024). The absorbance readings of the chlorophyll samples were taken at 664 and 647 nm through JENWAY 6505 UV/Vis spectrophotometer.

### 2.7 Statistical analysis

The results were analyzed using SPSS (IBM Corp., 2020), incorporating three biological replicates. Significance levels were indicated as (*) for P-values less than 0.05, (**) for P < 0.01, (***) for P < 0.001, and (****) for P < 0.0001, demonstrating the highest degree of significance.

## 3 Results

### 3.1 Illumina-based sequencing, *de novo* assembly, and functional annotation

Lately, the Illumina sequencing technology has emerged as a robust technique for genome analysis and discovery in non-model plants. In this research, transcriptome sequences were obtained from pooled leaves of Salvia guaranitica using the Illumina HiSeq 2500 platform. This process yielded approximately 8.2 Gb of raw data from the S. guaranitica leaves. Post-filtering and removal of adapter sequences, 38,521,658 reads (38.52 million) were obtained, containing 210,521,170 high-quality nucleotide bases. The quality metrics indicated that 94.95% of the bases had a quality score of Q20, 90.54% had a quality score of Q30, and the GC content was 48.58%. Our findings were consistent with previously obtained results of many other studies which utilized transcriptome tools to detect and identify key genes associated with various biomolecules in several species, including Vicia sativa, Dendrobium nobile, S. officinalis, Ocimum sanctum, Ocimum basilicum, and Cunninghamia lanceolata (Rastogi et al., 2014; Ali et al., 2017; Zhu et al., 2019; Huang et al., 2021; Zhang et al., 2023).

### 3.2 *De novo* assembly and transcriptome analysis

With respect to *de novo* assembly and transcriptome study, high-quality and pure reads were assembled using Trinity program (Ali et al., 2017, 2018; Mehmood et al., 2021). The assembly results yielded 200,298 RNA variants, the N50 length was 1,850 bp, the N90 length was 520 bp, and the mean length was 1,125 bp. Additionally, 75,100 unigenes were identified, with N50 equals to 1,524 bp, N90 is 320 bp, and a mean length of 965 bp. The assembled lengths were 200 to approximately 2,000 bp. The majority of transcripts (83,387 transcripts, 41.652%) were between 200 and 500 bp, then 48,252 transcripts (24.102%) between 1,000 and 2,000 bp, and 41,197 transcripts (20.578%) between 500 and 1,000 bp. Conversely, the fewest transcripts (27,462 transcripts, 13.731%) were longer than 2,000 bp. Similarly, the lengths of the unigene assemblies ranged from 200 to over 2,000 bp, with the majority (39,145 unigenes, 52.125%) between 200 and 500 bp, followed by 16,866 unigenes (22.458%) between 500 and 1,000 bp, and 13,837 unigenes (18.425%) between 1,000 and 2,000 bp. The fewest unigenes (5,251 unigenes, 6.992%) were longer than 2,000 bp. The length profile of the transcripts and unigenes is presented in **Supplementary Table S2**. Our findings correspond with results observed in other species, such as, Boehmeria nivea, Curcuma longa, M. sativa, S. officinalis, Centella asiatica, and Apium graveolens, where transcript and unigene lengths predominantly fell within 75 to 500 bp (Srividya et al., 2015; Zhu et al., 2019; Xiao et al., 2024).

### 3.3 Unigene annotation and classification

**Figure 1:**
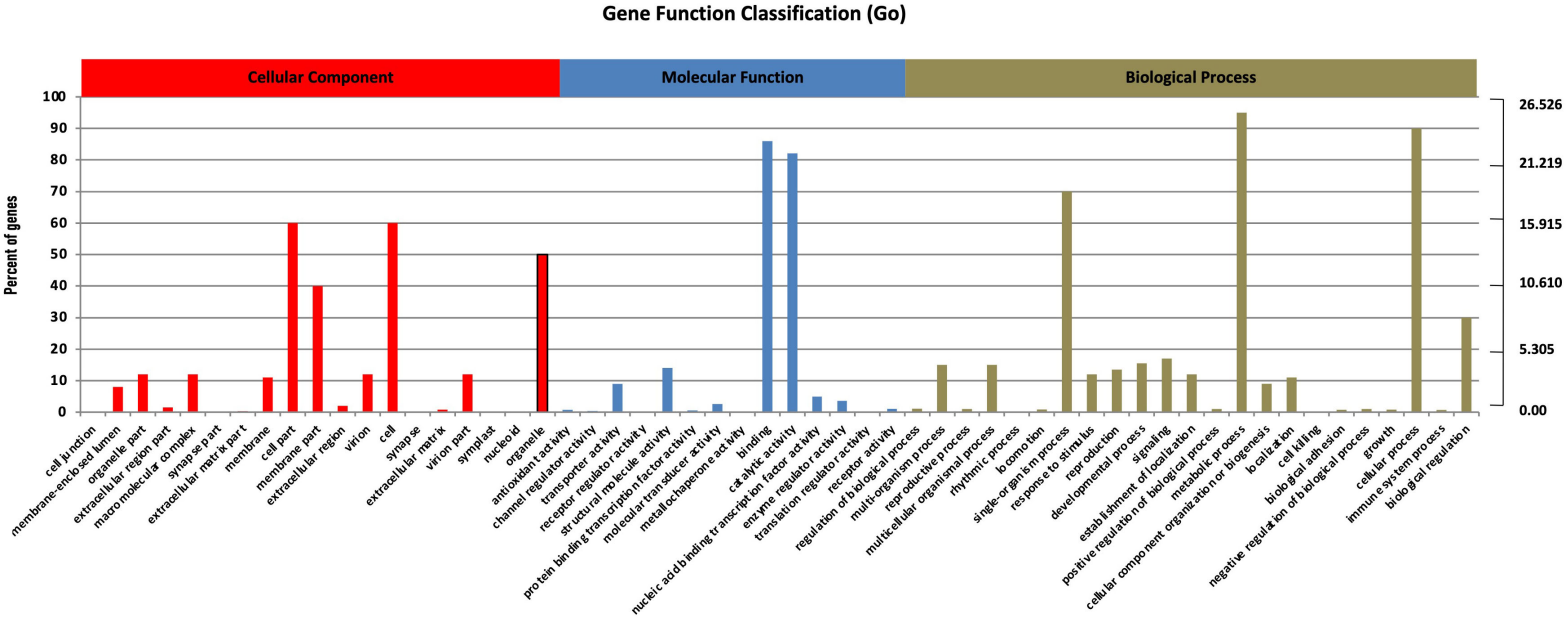
Unigenes annotations in *S. guaranitica*. The three main categories are BP, CC, and MF. In order to ascertain the biofunctions of the *S. guaranitica* transcriptome, KEGG pathways of the 75,100 unigenes were determined, with 11,746 unigenes (15.64%) ascribed to 270 pathways. Primarily, five major pathways were identified: **(A)** Cellular Processes, **(B)** Environmental Information Processing, **(C)** Genetic Information Processing, **(D)** Metabolism, and **(E)** Organismal Systems (**Figure 2**).

Nearly 75,100 unigenes served as search queries across NR (http://www.ncbi.nlm.nih.gov/), NT, KO, Swiss-Prot (http://www.ebi.ac.uk/uniprot/), PFAM, GO (http://www.geneontology.org/), and KEGG (https://www.kegg.jp/kegg/kegg2.html) **Supplementary Table S3**. The BLAST2GO program facilitated the sorting and ranking of the functions of all annotated unigenes, with 29,695 unigenes (39.54% of the assembled unigenes) assigned with at least one GO term. Inferred from the homology results, the unigenes were sorted into 56 functional groups across three main categories: 63,008 assigned to biological processes (BP), 48,517 to cellular components (CC), and 20,546 to molecular functions (MF). In the CC section, the most enriched GO terms were “cell part” (9,357) and “cell” (9,281). In the MF section, “binding” (14,258) and “catalytic activity” (12,754) were predominant. Within the BP section, “metabolic process” (15,326) and “cellular process” (14,820) were highly enriched (**Figure 1**). These findings are consistent with previous studies on the RNA profiles of S. miltiorrhiza, S. officinalis, O. sanctum, and O. basilicum, that also reported high percentages of these GO terms (Ali et al., 2017, 2018; Mehmood et al., 2021). 

**Figure 2:**
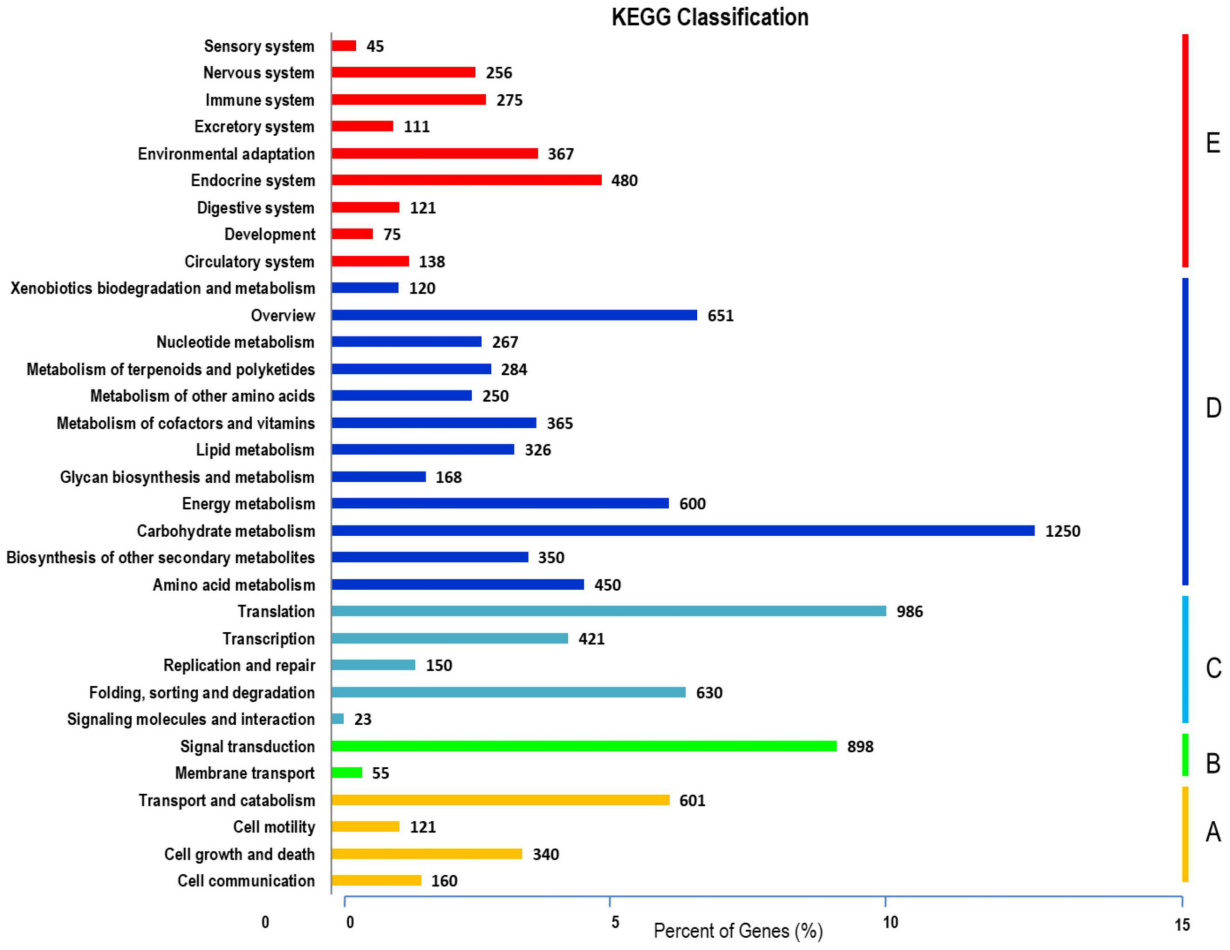
KEGG cellular processes pathways were classified into five largest categories; **(A)**, environmental information processing **(B)**, genetic information processing **(C)**, metabolism **(D)** and organismal systems **(E)**.

**Figure 3:**
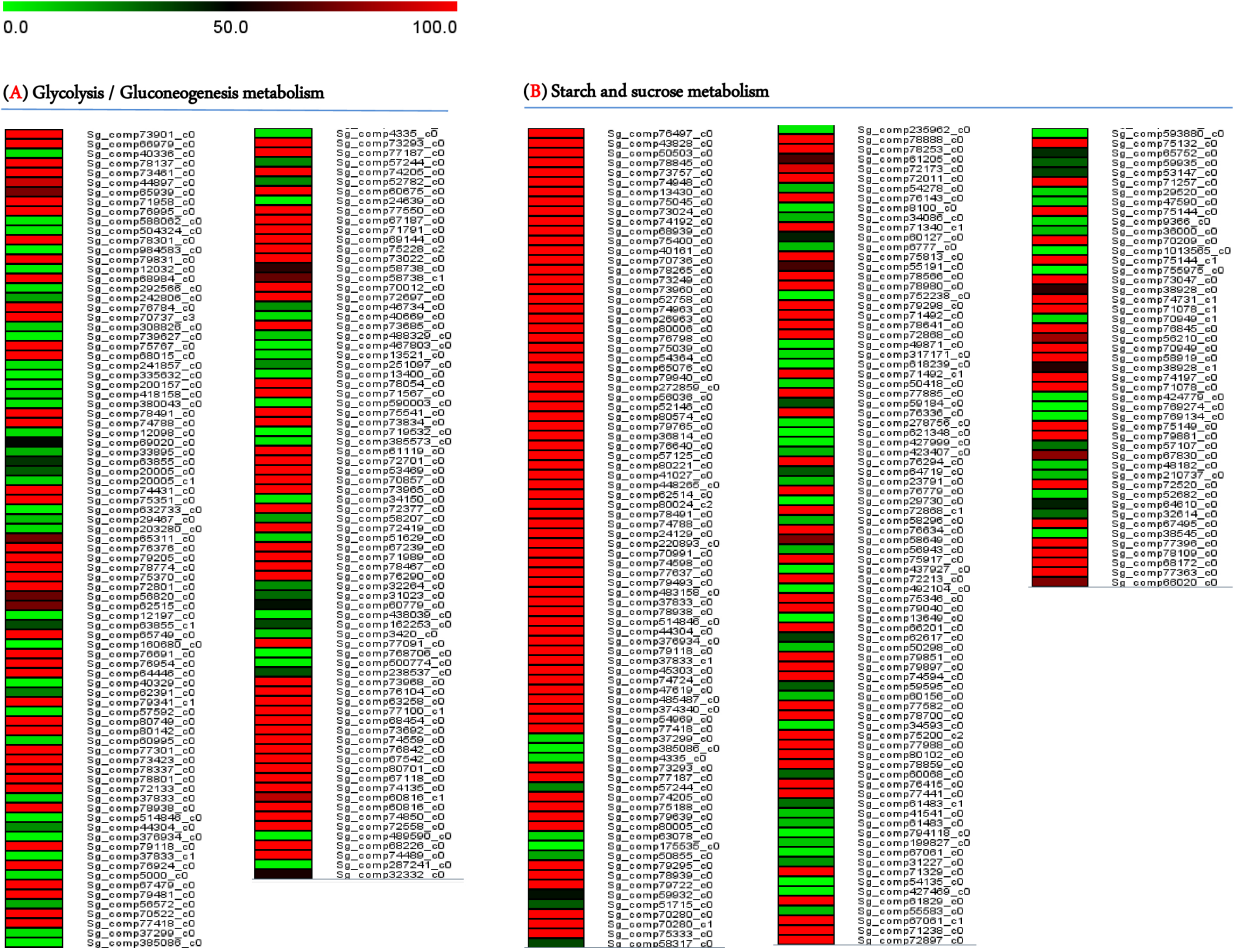
A heatmap illustrating the transcript levels of genes regulating **(A)** Glycolysis/Gluconeogenesis pathways and **(B)** Starch and Sucrose pathways.

Most transcripts belonged to the Metabolism section (5,081), subsequently, Genetic Information Processing (2,187), Organismal Systems (1,868), Cellular Processes (1,222), and Environmental Information Processing (976). From the data analysis, 1,250 transcripts were related to carbohydrate metabolism, with 410 putatively linked to starch, sucrose, and glycolysis metabolism. This included 175 genes involved in glycolysis/gluconeogenesis biosynthesis and 235 genes related to starch and sucrose biosynthesis. The levels of gene expressions were estimated through the UniProt database for annotation against the transcriptome libraries. Normalization and calculation were performed using the DESeq package (1.10.1), represented as fragments per kilobase of transcripts per million mapped fragments (FPKM) as shown in **Figure 3** and presented in **Supplementary Tables S4** and **S5**.

### 3.4 Patterns of tissue expressions, subcellular localizations and protein domain analysis

**Figure 4:**
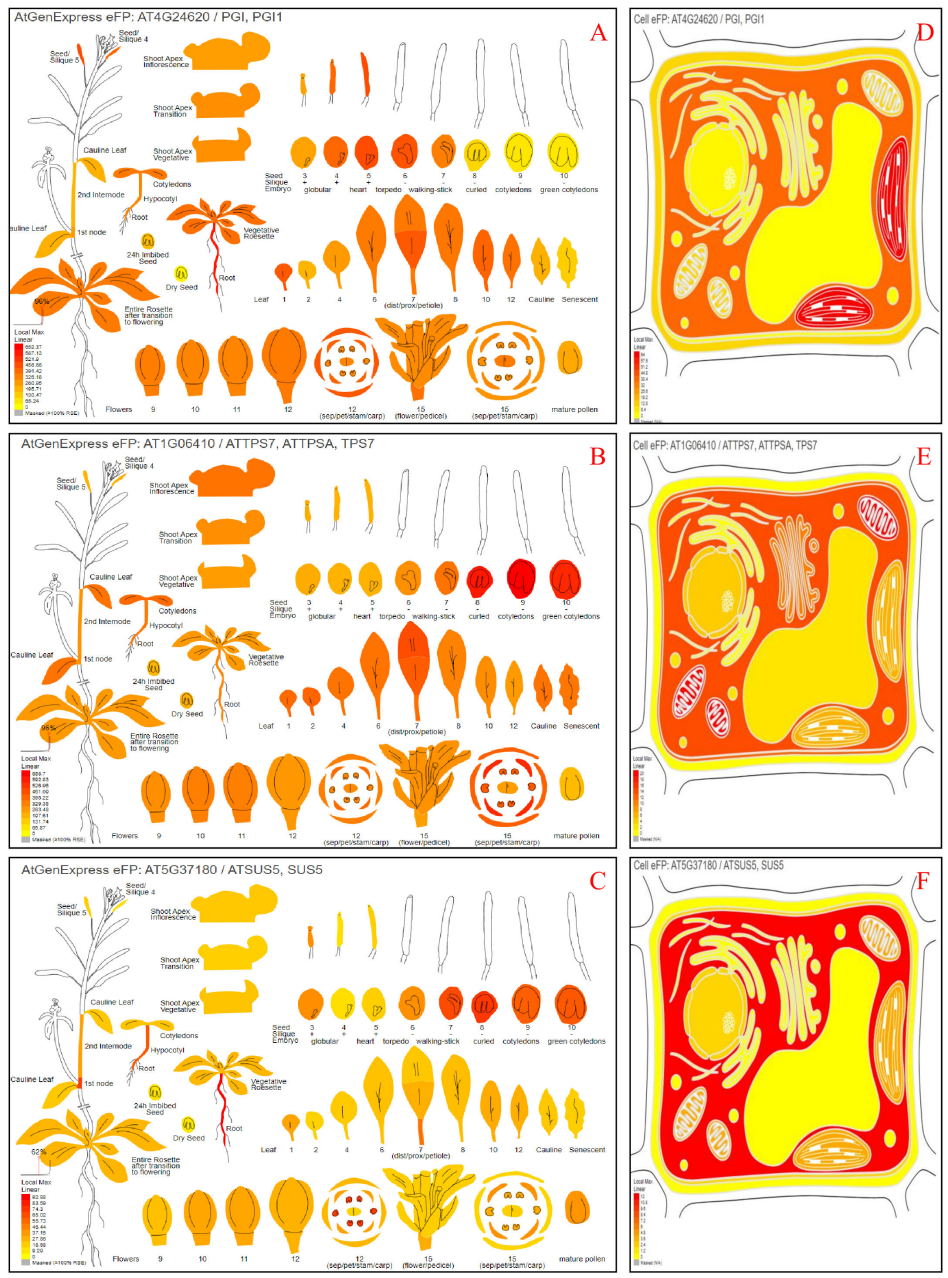
Depicting the potential an ‘electronic fluorescent pictograph’ website for investigating *A. thaliana* orthologous genes’ potential tissue expression and their proteins’ cellular localizations as retrieved from the eFPbrowsers (http://bar.utoronto.ca/efp/cgi-bin/efpWeb.cgi). Panels **(A, D)** demonstrate where cells may express SgGPI (AT4G24620). Panels **(B, E)** indicate where cells may express SgT6PS (AT1G06410). Panels **(C, F)** demonstrate where cells may express SgSUS (AT5G37180). The color box shows expression scale (greater red indicates more gene expression).

To identify the physiological roles of the SgGPI, SgT6PS, and SgSUS genes, we investigated their accumulation patterns across forty-seven tissues. The analysis was facilitated by the high similarity between SgGPI, SgT6PS, SgSUS, and the AT4G24620, AT1G06410, and AT5G37180 from *A. thaliana*, respectively. The relative expressions of SgGPI, SgT6PS, and SgSUS were observed in various tested tissues (**Figures 4A–C**). These results align with results reported (Ali et al., 2017, 2018, 2022a) who used similar tools in the BAR database to predict the putative expression patterns for several genes, such as, SoNEOD, SoHUMS, SoFLDH, SoLINS2, GmTPS21, SgTPSV, SgGERIS, and SgFARD, from S. officinalis, Glycine max, and S. guaranitica, respectively, showing heightened manifestation in leaves, roots, and seeds. Additionally, the localizations of SgGPI, SgT6PS, and SgSUS revealed that they are present in various cell organelles. For example, the SgGPI is predominantly located in the plastid, cytosol, extracellular space, and mitochondria. However, SgT6PS is mainly found in the mitochondrion, cytosol, plasma membrane, Golgi apparatus, nucleus, peroxisome, and vacuole. Meanwhile, SgSUS is primarily present in the cytosol, mitochondrion, plastid, nucleus, and plasma membrane (**Figures 4D–F**).

### 3.5 Quantitative RT-PCR analysis

**Figure 5:**
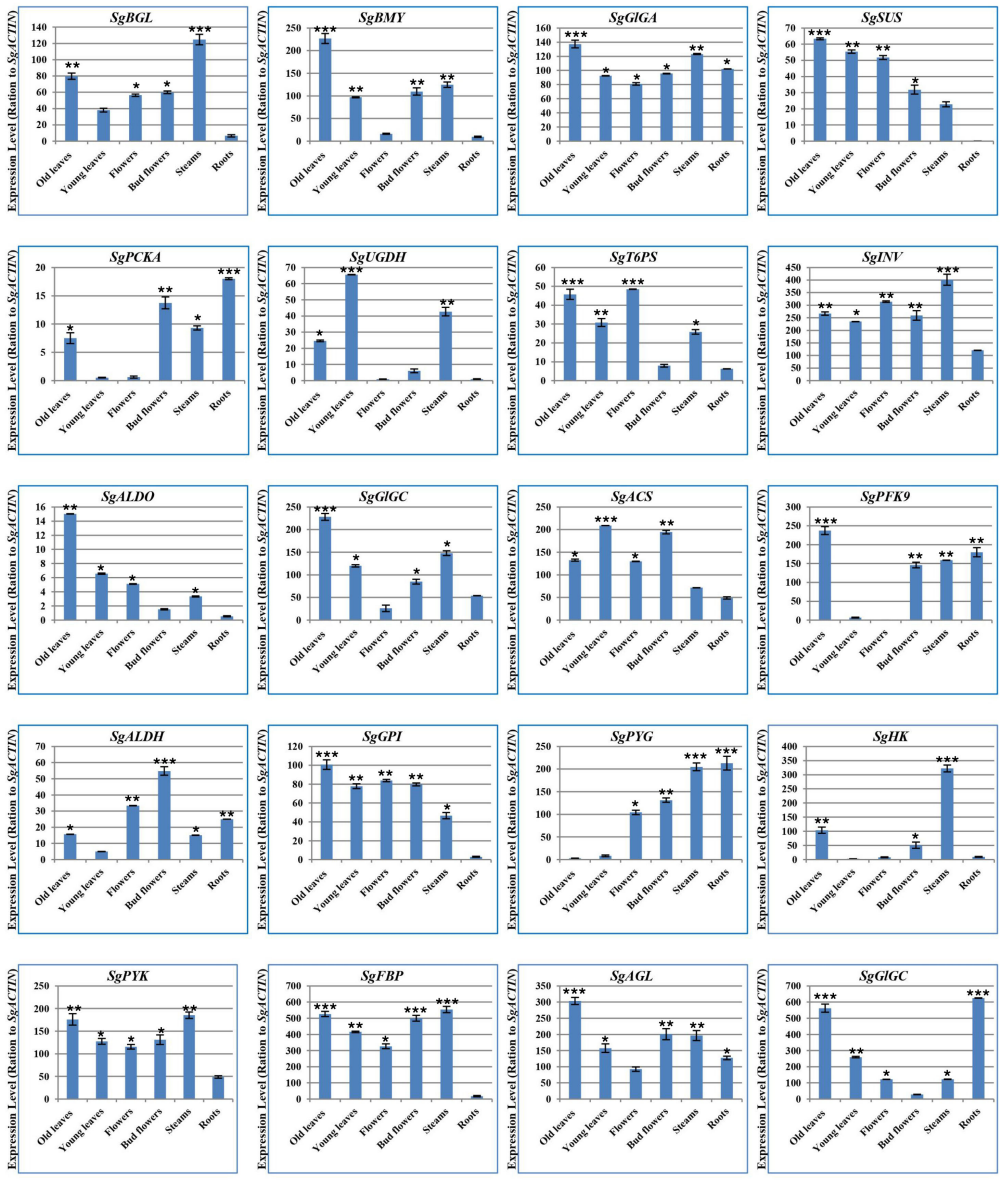
Quantitative RT-PCR of glycolysis/gluconeogenesis, starch and sucrose metabolism genes. The relative expressions of SgGPI, SgT6PS, SgSUS, SgPFK9, SgALDH, SgALDO, SgPYK, SgFBP, SgACS, SgPCKA, SgGlGA, SgGlGC, SgBMY, SgGBE1, SgAGL, SgBGL, SgHK, SgPYG, SgUGDH, and SgINV were calculated. The values are means ± SE of three biological replicates. Significance levels were indicated as (*) for P-values less than 5%, (**) for P < 1%, and (***) for P < 0.1%.

To elucidate the differential gene expression of glycolysis/gluconeogenesis, starch and sucrose biosynthesis genes (**Supplementary Table S6**; **Figure 5**) across different treatments, we employed the Bio-Rad Nucleic Acid Amplification and Detection systems (CFX384) with SYBR Green fluorescence and ROX as a passive reference dye (Newbio Industry, China), following protocols outlined in previous studies (Rastogi et al., 2014; Zhu et al., 2019; Huang et al., 2021; Santa Cruz et al., 2021). Primers were designed using the IDTdna tool (https://eu.idtdna.com/scitools/Applications/RealTimePCR/), as listed in **Supplementary Tables S6** and **S2**. The cycle threshold (CT) of the target genes was calculated using *SgB-ACTIN* as a reference gene to normalize gene expression levels. Relative gene expression levels were then determined through delta-delta C_t_ method (**Supplementary Tables S6**; **Figure 5**). The relative expression levels of several genes, including *SgGPI, SgT6PS, SgSUS, SgPFK9, SgALDH, SgALDO, SgPYK, SgFBP, SgACS, SgPCKA, SgGlGA, SgGlGC, SgBMY, SgGBE1, SgAGL, SgBGL, SgHK, SgPYG, SgUGDH*, and *SgINV*, were detected. For instance, *SgBMY, SgGlGA, SgSUS, SgALDO, SgGlGC, SgPFK9, SgGPI*, and *SgAGL* genes exhibited the top expression levels in mature leaves. In contrast, *SgUGDH* and *SgACS* genes showed the peak expression levels in immature leaves. Additionally, the *SgT6PS* gene demonstrated the maximum expression levels in flowers, whereas the *SgALDH* gene had the highest expression levels in flower buds. Moreover, *SgBGL, SgINV, SgHK, SgPYK*, and *SgFBP* genes demonstrated the peak levels in stems (**Figure 5**). Finally*, SgPCKA, SgPYG, and SgGlGC* genes were found with the greatest levels in roots. Interestingly, the qPCR analyses of these genes were in line with their expressions as measured by Illumina HiSeq 2500 (Hussain et al., 2017; Darwish et al., 2022; Ali et al., 2023b).

### 3.6 Phenotypic and functional characterization of *SgGPI*, *SgT6PS*, and *SgSUS* in *A. thaliana*

**Figure 6:**
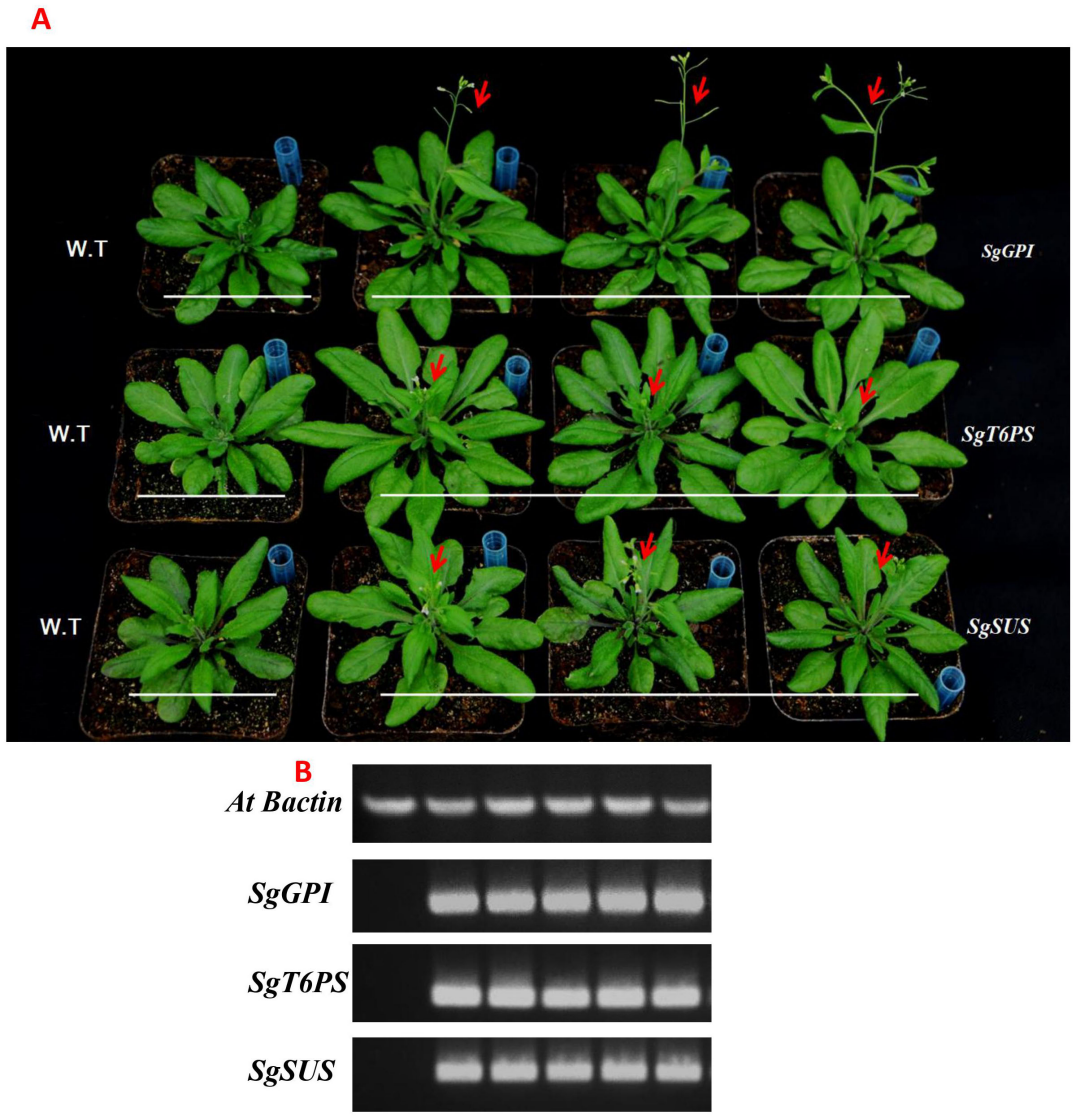
Overexpression of the *SgGPI*, *SgT6PS* and *SgSUS* genes from *S. guaranitica* in transgenic *A. thaliana*. **(A)** Comparison of the phenotypes of the transgenic *A.. thaliana* and wild type *A. thaliana*. **(B)** Semi-qRT-PCR of the genes regulating starch, sucrose, and glycolysis biosynthesis.

To further investigate the biological function of *SgGPI, SgT6PS*, and *SgSUS*, we transferred the overexpression vector pB2GW7-*SgGPI*, pB2GW7-*SgT6PS* and pB2GW7-*SgSUS* directed by 35S promoter into *A. thaliana*. We obtained twelve homozygous transgenic lines from each transformed gene and the successful transformed lines was confirmed by PCR and semi-qRT-PCR analysis (**Figures 6A, B**). Compared to the WT, a significant difference in leaf development and plant growth were observed in all lines overexpressing these genes (**Figure 6A**). Interestingly, the flowering process of transgenic *A. thaliana* was accelerated earlier than that of W.T plants (**Figure 6A**).

### 3.7 Overexpression of SgGPI, SgT6PS and SgSUS alters various physiological and biochemical attributes in transgenic plants

**Figure 7:**
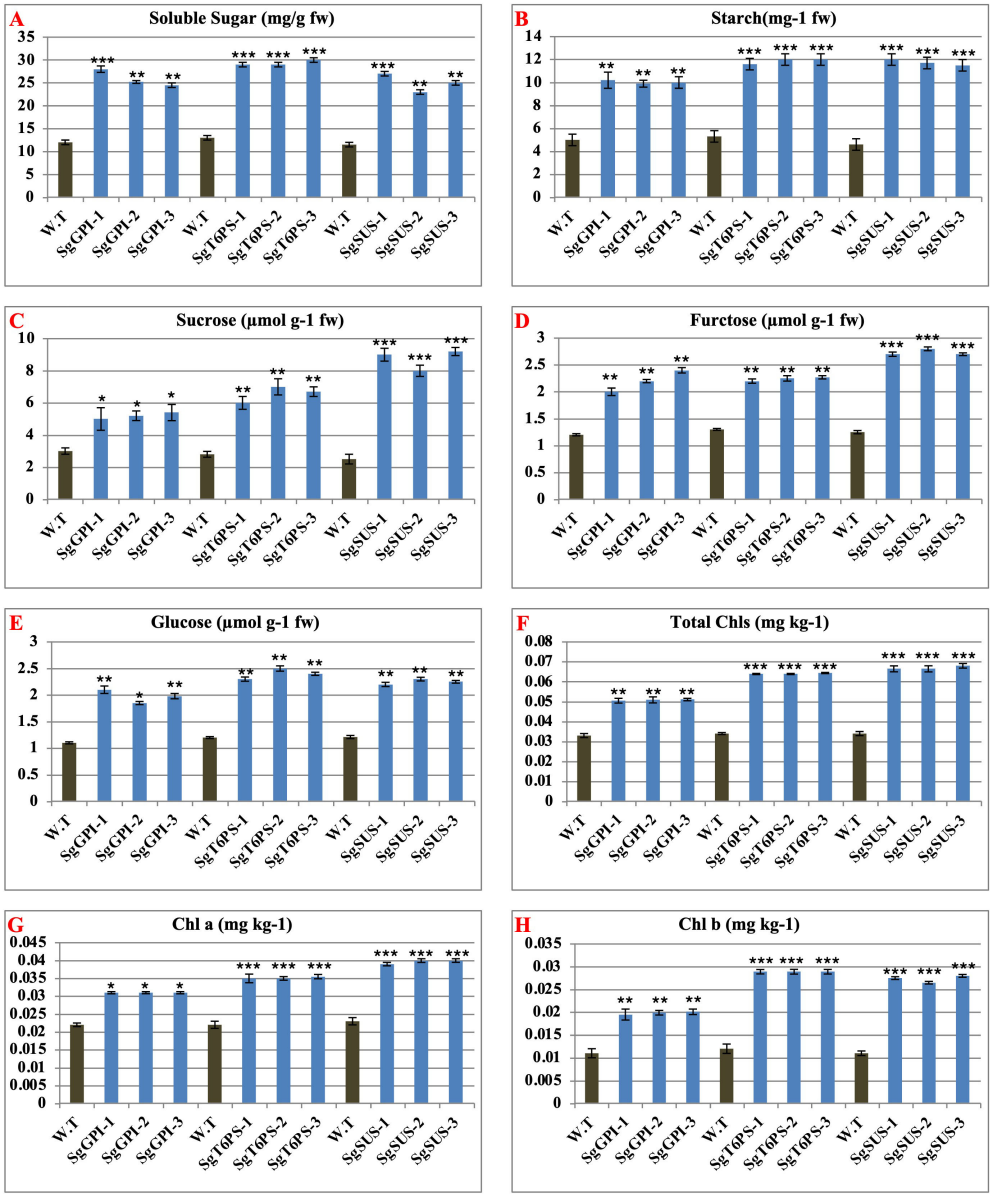
Analysis of physiological and biochemical parameters from wild and transgenic *A thaliana* under the effects of overexpression of SgGPI, SgT6PS and SgSUS separately. **(A)** soluble sugar; **(B)** starch; **(C)** sugar; **(D) **fructose; **(E)** glucose; **(F)** total chlorophyll; **(G)** chlorophyll a; **(H)** chlorophyll (b) Significance levels were indicated as (*) for P-values less than 0.05, (**) for P < 0.01, and (***) for P < 0.001.

Sugars serve as the primary carbon source for synthesizing various pathways involved in both primary and secondary metabolites. The levels of soluble sugars were assessed in transgenic *A. thaliana* plants overexpressing SgGPI, SgT6PS, and SgSUS, revealing significant increases compared to the wild-type (WT). Furthermore, starch, total sugar, glucose, and fructose contents were quantified in these transgenic plants. Results indicated higher levels of these components in all transgenic lines compared to WT (**Figure 7**). Specifically, the average starch content increased by approximately 5.033-fold (10.033/5.0) for *A. thaliana* plants overexpressing SgGPI, 6.566-fold (11.866/5.3) for *A. thaliana* plants overexpressing SgT6PS, and 7.133-fold (11.700/4.6) for A. thaliana plants overexpressing SgSUS compared to WT. Similarly, average sugar contents visually increased by 2.2-fold (5.2/3) for *A. thaliana* plants overexpressing SgGPI, 3.766-fold (6.566/2.8) for *A. thaliana* plants overexpressing SgT6PS, and 6.233-fold (8.733/2.5) for *A. thaliana* plants overexpressing SgSUS. Glucose levels showed respective increases of 0.87-fold (1.97/1.1), 1.2-fold (2.4/1.2), and 1.04-fold (2.25/1.21), while fructose levels increased by 1.0-fold (2.2/1.2), 0.94-fold (2.24/1.3), and 1.52-fold (2.733/1.25) for *A. thaliana* plants overexpressing SgGPI, *A. thaliana* plants overexpressing SgT6PS, and A. thaliana plants overexpressing SgSUS compared to WT.

Additionally, the total chlorophyll, chlorophyll a, and chlorophyll b contents were evaluated in these transgenic plants. Results indicated higher levels of these chlorophyll components in all transgenic lines compared to WT (**Figure 7**). For instance, the average total chlorophyll content increased by 0.017-fold (0.0508/0.033) for *A. thaliana* plants overexpressing SgGPI, 0.030-fold (0.0640/0.034) for *A. thaliana* plants overexpressing SgT6PS, and 0.033-fold (0.067/0.034) for *A. thaliana* plants overexpressing SgSUS compared to WT. Similarly, average chlorophyll a level increased by 0.009-fold (0.031/0.022) for *A. thaliana* plants overexpressing SgGPI, 0.0131-fold (0.0351/0.022) for *A. thaliana* plants overexpressing SgT6PS, and 0.016-fold (0.039/0.023) for *A. thaliana* plants overexpressing SgSUS, while chlorophyll b levels increased by 0.068-fold (0.0795/0.011) for *A. thaliana* plants overexpressing SgGPI, 0.0747-fold (0.0867/0.012) for *A. thaliana* plants overexpressing SgT6PS, and 0.071-fold (0.082/0.011) for *A. thaliana* plants overexpressing SgSUS compared to WT.

## 4 Discussion

Carbohydrate biosynthesis is the primary driver of plant growth and development. Carbohydrates are highly susceptible to biotic and abiotic stresses, which influence yield parameters. Therefore, identifying genes that regulate carbohydrate production in *S. guaranitica* will facilitate the development of targeted breeding techniques to enhance desirable traits such as growth, stress resilience, and medicinal properties. *S. guaranitica* is renowned for its rich array of primary and secondary metabolites, including disaccharides, starch, terpenoids, and flavonoids. Disaccharides, found across various organisms, play diverse roles in plant growth, development, and resistance to environmental stresses through pathways like glycolysis/gluconeogenesis and starch/sucrose metabolism, which share common enzymes and substrates, illustrating their interdependence. This study employed RNA-Seq technology to identify key enzymes in these pathways from *S. guaranitica* leaf transcriptomes, revealing over 75,100 unigenes encompassing all metabolic pathways. Gene ontology analysis highlighted 410 genes potentially involved in starch, sucrose, and glycolysis metabolism, including 175 related to glycolysis/gluconeogenesis and 235 to starch/sucrose biosynthesis, as illustrated **Figure 3** and detailed in **Supplementary Tables S4** and **S5**. These findings underscore RNA-Seq’s efficacy in elucidating mechanisms underlying metabolite synthesis (Liu et al., 2020; De Vega et al., 2021; Yang et al., 2023).

Functional characterization of *SgGPI*, *SgT6PS*, and *SgSUS* through putative tissue expression patterns, subcellular localization, and protein domain analysis using tools like eFP browsers, Cell-eFP browsers, and InterPro database enhances our understanding of their roles and expression levels see **Figure 4**. *In silico* analyses validate their predicted functions, excluding irrelevant pathways, and affirm their significance in plant biology. Furthermore, these previous tools were used in various studies such as; (Ali et al., 2022a, 2022c, 2022b, 2023b, 2023a; Abdelhameed et al., 2024b, 2024a).

In general, the expression levels of multiple genes which encoded glycolysis/gluconeogenesis, starch and sucrose enzymes (e.g. *SgGPI, SgT6PS, SgSUS, SgPFK9, SgALDH, SgALDO, SgPYK, SgFBP, SgACS, SgPCKA, SgGlGA, SgGlGC, SgBMY, SgGBE1, SgAGL, SgBGL, SgHK, SgPYG, SgUGDH* and *SgINV*) were detected at different tissues especially in leaves, and these previous genes may effectively enhance the entire pathways **Figure 5**. Moreover, any changes in the expression profiles of the aforementioned genes are often bound to the accumulation levels of biochemical contents from soluble sugar, starch, sugar, fructose, glucose, total chlorophyll, chlorophyll a and chlorophyll b. Also, these changes in the genes expression levels and their relationship to the accumulation of previous compounds explain the extent of the plant’s response to growth and the increase in the plant growth and plant biomass (Sung et al., 1988; Stein and Granot, 2019; Li et al., 2020; Taujale et al., 2021).

In comparison to control plants (non-transgenic), overexpression of *SgGPI, SgT6PS* and *SgSUS* controlled under 35S promoter caused manifest difference in phenotypic traits such as; plant biomass, leaves area, leaves number and early flowering (**Figure 6**). The results describe that transformation of previous genes under 35S promoter is expressed in whole transgenic *A. thaliana* plants as we confirmed by semi-qRT-PCR data, and this expression is related with vegetative growth and early flowering formation as well. The current findings and results are in close agreement with previous investigations by (Zhang et al., 2016; Ahmed et al., 2020; Liu et al., 2020; De Vega et al., 2021) which study the roles of various glycolysis, starch and sucrose genes from different plants (e.g., *Allium cepa* L., *Sorghum*, *Taro Corm*, *Miscanthus hybrids* and *Castanea henryi*) in plant growth and increase of biomass yield. Through our previous results, we can infer that *SgGPI, SgT6PS* and *SgSUS* encode essential enzymes involved in *S. guaranitica* development, as the activity of these enzymes are correlated with plant growth and increase of biomass yield.

Furthermore, this study evaluated changes in physiological and biochemical traits (soluble sugars, starch, sugars, fructose, glucose, total chlorophyll, chlorophyll a, and chlorophyll b) in wild-type and transgenic *A. thaliana* plants overexpressing *SgGPI, SgT6PS*, and *SgSUS*, as shown in **Figure 7**. These traits directly influence physiological and morphological variations that impact plant growth, consistent with earlier findings by (Dian et al., 2003, 2005; Akihiro et al., 2005; Wang and Ruan, 2013; Ahmed et al., 2018, 2020).

In conclusion, our study contributes valuable information to the limited transcriptome resources of *Salvia*, one of the largest genera in the Lamiaceae families, known for its diverse specialized secondary and primary metabolites. The *S. guaranitica* transcriptome data we provide here should be valuable for metabolic engineering, fundamental biological research, and plant improvement programs. Transgenic *A. thaliana* lines with high phenotypic traits and biochemical contents generated in this study should serve as a useful adjunct in enhancing the maximize the benefit of these genes in improving plants and increasing production.

## 5 Conclusions

In this study, we employed NGS technology to generate high-quality transcriptome data from *S. guaranitica* leaves. Utilizing *de novo* sequencing and analysis tools, we assembled data obtained from the Illumina HiSeq 2500 system to characterize and identify genes associated with starch, sucrose, and glycolysis biosynthesis pathways. The transcriptome analysis revealed numerous genes encoding enzymes pivotal to these metabolic pathways in *S. guaranitica*. Specifically, we conducted cloning and bioinformatics analyses of *S. guaranitica. SgGPI, SgT6PS*, and *SgSUS*. Our findings confirm that these genes encode prototypical GPI, T6PS, and SUS proteins. Overexpression of *SgGPI, SgT6PS*, and *SgSUS* in *A. thaliana* resulted in enhanced leaf growth, plant development, and accelerated flowering. Furthermore, these genes significantly increased soluble sugar, starch, sugars (glucose and fructose), total chlorophyll, chlorophyll a, and chlorophyll b contents in transgenic tobacco lines compared to wild-type (WT). Overall, our study provides insights into the regulatory mechanisms of *SgGPI, SgT6PS*, and *SgSUS* and their roles in starch, sucrose, and glycolysis metabolism pathways. These findings open new avenues for biotechnological applications utilizing these genes.

## Data Availability

The original contributions presented in the study are publicly available. This data can be found here: NCBI, PRJNA1163088.
