# Supplementary material for: Harnessing de novo transcriptome sequencing to identify and characterize genes regulating carbohydrate biosynthesis pathways in Salvia guaranitica L
Source: Front Plant Sci. 2024 Sep 26;15:1467432. doi: 10.3389/fpls.2024.1467432 (PMC11464306; doi:10.3389/fpls.2024.1467432)
Supplement: Supplementary file 1 [file Table1.pdf]

Supplementary Material

Table S1. Sage genes and primers employed for full-length glycolysis/gluconeogenesis, starch, and sucrose metabolism cDNAs.

S. guaranitica ID	Gene name	Primer name	Short primer sequence	Long primer sequence	Melting Temp. (ºC)	Size (bp)
**DSC|comp74788_c0**	*SgGPI*	PCR- *SgGPI* -F	ATGGCTTCCTCCGTCTCCG	GGGGACAAGTTTGTACAAAAAAGCAGGCTTCATGGCTTCCTCCGTCT	60	1823
		PCR- *SgGPI* -R	TAGGGGTACATATCATCAATATTGCAC	GGGGACCACTTTGTACAAGAAAGCTGGGTTAGGGGTACATATCATCA		
**DSC|comp79765_c0**	*SgT6PS*	PCR-*SgT6PS*-F	ATGATGTCAAGATCTTATACCAACCT	GGGGACAAGTTTGTACAAAAAAGCAGGCTTCATGATGTCAAGATCTTAT	60	2604
		PCR-*SgT6PS*-R	TTAAACAAATGAAGTAACACGGC	GGGGACCACTTTGTACAAGAAAGCTGGGTTTAAACAAATGAAGTAAC		
**DSC|comp77396_c0**	*SgSUS*	PCR-*SgSUS*-F	ATGGCAGCAGCATTGAAGAGA	GGGGACAAGTTTGTACAAAAAAGCAGGCTTCATGGCAGCAGCATTGA	60	2497
		PCR-*SgSUS*-R	ATAACTTCTGCAATGTGGACTGAGA	GGGGACCACTTTGTACAAGAAAGCTGGGTATAACTTCTGCAATGT		
