# Supplementary material for: Harnessing de novo transcriptome sequencing to identify and characterize genes regulating carbohydrate biosynthesis pathways in Salvia guaranitica L
Source: Front Plant Sci. 2024 Sep 26;15:1467432. doi: 10.3389/fpls.2024.1467432 (PMC11464306; doi:10.3389/fpls.2024.1467432)
Supplement: Supplementary file 2 [file Table2.pdf]

Supplementary Material

Table S2. Transcript and UniGene length variability in the *S. guaranitica* transcriptome.

Nucleotide length (bp)	Number of transcripts	Number of UniGenes
**200-500bp**	83.387 (41.652%)	39.145 (52.125 %)
**500-1kbp**	41.197 (20.578%)	16.866 (22.458 %)
**1k-2kbp**	48.252 (24.102%)	13.837 (18.425 %)
**>2kbp**	27.462 (.13731%)	5.251 (6.992 %)
**Total**	200.298	75.100
**Min Length (bp)**	201	201
**Mean Length (bp)**	1.125	965
**Median Length (bp)**	859	377
**Max Length (bp)**	14.847	14.847
**N50 (bp)**	1.88350	1.524
**N90 (bp)**	520	320
**Total Nucleotides length (bp)**	210,521,170	69.081.039
