# Supplementary material for: Harnessing de novo transcriptome sequencing to identify and characterize genes regulating carbohydrate biosynthesis pathways in Salvia guaranitica L
Source: Front Plant Sci. 2024 Sep 26;15:1467432. doi: 10.3389/fpls.2024.1467432 (PMC11464306; doi:10.3389/fpls.2024.1467432)
Supplement: Supplementary file 3 [file Table3.pdf]

Supplementary Material

Table S3. Functional annotations of the *S. guaranitica* transcriptome.

Annotation database	No. of UniGene hits	Percentage %
Annotated in NR	36.762	48.95
Annotated in NT	13.871	18.47
Annotated in KO	11.746	15.64
Annotated in Swiss-Prot	27.126	36.12
Annotated in PFAM	25.766	34.31
Annotated in GO	29.695	39.54
Annotated in KOG	14.239	18.96
Annotated in all Databases	5.467	7.28
Annotated in at least one Database	40.126	53.43
Total UniGenes	75.100	100
