# Supplementary material for: Harnessing de novo transcriptome sequencing to identify and characterize genes regulating carbohydrate biosynthesis pathways in Salvia guaranitica L
Source: Front Plant Sci. 2024 Sep 26;15:1467432. doi: 10.3389/fpls.2024.1467432 (PMC11464306; doi:10.3389/fpls.2024.1467432)
Supplement: Supplementary file 4 [file Table4.pdf]

Supplementary Material

Table S4. Transcript abundance of glycolysis/gluconeogenesis biosynthesis genes as per the *S. guaranitica* transcriptome data annotation.

Pathway / ***Glycolysis/Gluconeogenesis***						
Gene Name	**KEGG Entry**	**UniGene ID**	**EC. No.**	**Gene Length**	**Reads in Leaf**	**FPKM**
6-phosphofructokinase 1	K00850	Sg_comp594855_c0	2.7.1.11	309	3	1.29
6-phosphofructokinase 1	K00850	Sg_comp73901_c0	2.7.1.11	555	1101.97	31.33
6-phosphofructokinase 1	K00850	Sg_comp66979_c0	2.7.1.11	1386	895.38	28.37
6-phosphofructokinase 1	K00850	Sg_comp40336_c0	2.7.1.11	237	6	6.52
6-phosphofructokinase 1	K00850	Sg_comp78137_c0	2.7.1.11	1572	1422.31	42.73
6-phosphofructokinase 1	K00850	Sg_comp73461_c0	2.7.1.11	1710	205.99	7.34
6-phosphofructokinase 1	K00850	Sg_comp44897_c0	2.7.1.11	375	90	7.04
6-phosphofructokinase 1	K00850	Sg_comp65939_c0	2.7.1.11	984	74	8.73
Enolase	K01689	Sg_comp71958_c0	4.2.1.11	477	833.73	21.56
Enolase	K01689	Sg_comp76995_c0	4.2.1.11	1542	1359.59	38.18
Enolase	K01689	Sg_comp588062_c0	4.2.1.11	315	4	1.6
Enolase	K01689	Sg_comp504324_c0	4.2.1.11	309	4	0.92
Enolase	K01689	Sg_comp78301_c0	4.2.1.11	1353	7371.45	252.95
Enolase	K01689	Sg_comp984583_c0	4.2.1.11	249	1	0.87
Aldehyde dehydrogenase family 7 member A1	K14085	Sg_comp79831_c0	1.2.1.3,1 1.2.1.8 1.2.1.3	1623	2785.73	115.91
Fructose-bisphosphatealdolase, class I	K01623	Sg_comp12032_c0	4.1.2.13	222	3	2.78
Fructose-bisphosphatealdolase, class I	K01623	Sg_comp68984_c0	4.1.2.13	1212	76188.04	2695.28
Fructose-bisphosphatealdolase, class I	K01623	Sg_comp292566_c0	4.1.2.13	315	5	1.98
Fructose-bisphosphatealdolase, class I	K01623	Sg_comp242806_c0	4.1.2.13	594	18	1.75
Fructose-bisphosphatealdolase, class I	K01623	Sg_comp76784_c0	4.1.2.13	1104	2067.98	67.56
Fructose-bisphosphatealdolase, class I	K01623	Sg_comp70737_c3	4.1.2.13	1056	29673.79	1207.01
Fructose-bisphosphatealdolase, class I	K01623	Sg_comp308826_c0	4.1.2.13	294	9	1.51
Fructose-bisphosphatealdolase, class I	K01623	Sg_comp739627_c0	4.1.2.13	195	2	0.96
Fructose-bisphosphatealdolase, class I	K01623	Sg_comp75767_c0	4.1.2.13	1203	6062.11	390.76
Fructose-bisphosphatealdolase, class I	K01623	Sg_comp68015_c0	4.1.2.13	831	2686.11	156.05
Fructose-bisphosphatealdolase, class I	K01623	Sg_comp241857_c0	4.1.2.13	204	5	11.93
Fructose-bisphosphatealdolase, class I	K01623	Sg_comp335632_c0	4.1.2.13	333	1	0.35
Fructose-bisphosphatealdolase, class I	K01623	Sg_comp200157_c0	4.1.2.13	378	2	0.52
Fructose-bisphosphatealdolase, class I	K01623	Sg_comp418158_c0	4.1.2.13	318	7	1.47
Fructose-bisphosphatealdolase, class II	K01624	Sg_comp380043_c0	4.1.2.13	339	5	1.26
glucose-6-phosphate isomerase	K01810	Sg_comp78491_c0	5.3.1.9	1827	2156.03	62.11
Glucose-6-phosphate isomerase	K01810	Sg_comp74788_c0	5.3.1.9	1833	3448.56	77.2
Pyruvate kinase	K00873	Sg_comp12098_c0	2.7.1.40	300	7	1.55
Pyruvate kinase	K00873	Sg_comp69020_c0	2.7.1.40	618	49	7.61
Pyruvate kinase	K00873	Sg_comp33895_c0	2.7.1.40	234	15	3.92
Pyruvate kinase	K00873	Sg_comp63855_c0	2.7.1.40	366	40	11.22
Pyruvate kinase	K00873	Sg_comp20005_c0	2.7.1.40	384	32	8.08
Pyruvate kinase	K00873	Sg_comp20005_c1	2.7.1.40	228	9	11.65

Gene Name	KEGG Entry	UniGene ID	EC. No.	Gene Length	Reads in Leaf	FPKM
Pyruvate kinase	K00873	Sg_comp74431_c0	2.7.1.40	2433	1985.04	99.89
Pyruvate kinase	K00873	Sg_comp75351_c0	2.7.1.40	1590	2310.69	124.54
Pyruvate kinase	K00873	Sg_comp632733_c0	2.7.1.40	249	2	1.74
Pyruvate kinase	K00873	Sg_comp29467_c0	2.7.1.40	234	12	13.79
Pyruvate kinase	K00873	Sg_comp203280_c0	2.7.1.40	201	7	17.72
Pyruvate kinase	K00873	Sg_comp65311_c0	2.7.1.40	411	73	16.03
Pyruvate kinase	K00873	Sg_comp76376_c0	2.7.1.40	381	6062.69	164.37
Pyruvate kinase	K00873	Sg_comp79205_c0	2.7.1.40	492	594.44	48.56
Pyruvate kinase	K00873	Sg_comp78774_c0	2.7.1.40	1908	2941.94	80.7
Pyruvate kinase	K00873	Sg_comp75370_c0	2.7.1.40	207	1216.22	29.17
Pyruvate kinase	K00873	Sg_comp72801_c0	2.7.1.40	1065	1010.86	59.87
Pyruvate kinase	K00873	Sg_comp56820_c0	2.7.1.40	624	71	5.36
Pyruvate kinase	K00873	Sg_comp62515_c0	2.7.1.40	669	73	8.04
Pyruvate kinase	K00873	Sg_comp12197_c0	2.7.1.40	300	2	0.93
Pyruvate kinase	K00873	Sg_comp63855_c1	2.7.1.40	309	35	7.47
Pyruvate kinase	K00873	Sg_comp65749_c0	2.7.1.40	462	148	25.98
Pyruvate kinase	K00873	Sg_comp160680_c0	2.7.1.40	207	0	0
Pyruvate kinase	K00873	Sg_comp76691_c0	2.7.1.40	1875	2580.75	58.82
Pyruvate kinase	K00873	Sg_comp76954_c0	2.7.1.40	1761	603.17	33.93
Pyruvate dehydrogenase E2 component (dihydrolipoamideacetyltransferase)	K00627	Sg_comp64446_c0	2.3.1.12	552	197.05	25.02
Pyruvate dehydrogenase E2 component (dihydrolipoamideacetyltransferase)	K00627	Sg_comp40329_c0	2.3.1.12	261	1	0.73
Pyruvate dehydrogenase E2 component (dihydrolipoamideacetyltransferase)	K00627	Sg_comp62391_c0	2.3.1.12	141	25	3.96
Pyruvate dehydrogenase E2 component (dihydrolipoamideacetyltransferase)	K00627	Sg_comp79341_c1	2.3.1.12	1476	1252.1	71.79
Pyruvate dehydrogenase E2 component (dihydrolipoamideacetyltransferase)	K00627	Sg_comp57592_c0	2.3.1.12	201	3	7.16
Pyruvate dehydrogenase E2 component (dihydrolipoamideacetyltransferase)	K00627	Sg_comp80749_c0	2.3.1.12	1944	720.87	25.12
Pyruvate dehydrogenase E2 component (dihydrolipoamideacetyltransferase)	K00627	Sg_comp80142_c0	2.3.1.12	1677	1247.33	35.7
Pyruvate dehydrogenase E2 component (dihydrolipoamideacetyltransferase)	K00627	Sg_comp60995_c0	2.3.1.12	90	7	4.63
Fructose-1,6-bisphosphatase I	K03841	Sg_comp77301_c0	3.1.3.11	774	4842.75	179.55
Fructose-1,6-bisphosphatase I	K03841	Sg_comp73423_c0	3.1.3.11	1236	7011.19	264.11
Fructose-1,6-bisphosphatase I	K03841	Sg_comp78337_c0	3.1.3.11	1251	873.3	35.48
Fructose-1,6-bisphosphatase I	K03841	Sg_comp78801_c0	3.1.3.11	1242	2034.72	141.66
Glyceraldehyde-3-phosphate dehydrogenase (NADP+)	K00131	Sg_comp72133_c0	1.2.1.9	1497	11529.04	370.22
Phosphoglucomutase	K01835	Sg_comp37833_c0	5.4.2.2	291	7	3.62
Phosphoglucomutase	K01835	Sg_comp78938_c0	5.4.2.2	1953	6893.07	214.26
Phosphoglucomutase	K01835	Sg_comp514846_c0	5.4.2.2	219	1	1.5
Phosphoglucomutase	K01835	Sg_comp44304_c0	5.4.2.2	207	22	46.89
Phosphoglucomutase	K01835	Sg_comp376934_c0	5.4.2.2	225	1	1.35
Phosphoglucomutase	K01835	Sg_comp79118_c0	5.4.2.2	1758	2920.12	255.54
Phosphoglucomutase	K01835	Sg_comp37833_c1	5.4.2.2	300	2	0.91
2,3-bisphosphoglycerate-dependent phosphoglyceratemutase	K01834	Sg_comp76924_c0	5.4.2.11	1047	439.03	18.8
2,3-bisphosphoglycerate-dependent phosphoglyceratemutase	K01834	Sg_comp5000_c0	5.4.2.11	213	5	2.29
2,3-bisphosphoglycerate-dependent phosphoglyceratemutase	K01834	Sg_comp67479_c0	5.4.2.11	867	175	7.21
Acetyl-CoA synthetase	K01895	Sg_comp79481_c0	6.2.1.1	2490	3396.5	61.06
Acetyl-CoA synthetase	K01895	Sg_comp56572_c0	6.2.1.1	249	16	11.48

Gene Name	KEGG Entry	UniGene ID	EC. No.	Gene Length	Reads in Leaf	FPKM
L-lactate dehydrogenase	K00016	Sg_comp70522_c0	1.1.1.27	1086	240	10.82
Hexokinase	K00844	Sg_comp77418_c0	2.7.1.1	1587	174	4.32
Hexokinase	K00844	Sg_comp37299_c0	2.7.1.1	426	6	1.22
Hexokinase	K00844	Sg_comp385086_c0	2.7.1.1	222	2	2.49
Hexokinase	K00844	Sg_comp4335_c0	2.7.1.1	300	5	2.36
Hexokinase	K00844	Sg_comp73293_c0	2.7.1.1	258	665.14	35.2
Hexokinase	K00844	Sg_comp77187_c0	2.7.1.1	1536	2010.24	49.58
Hexokinase	K00844	Sg_comp57244_c0	2.7.1.1	273	23	4.11
Hexokinase	K00844	Sg_comp74205_c0	2.7.1.1	1266	146.47	8.63
Glucose-6-phosphate 1-epimerase	K01792	Sg_comp52782_c0	5.1.3.15	255	23	18.24
Glucose-6-phosphate 1-epimerase	K01792	Sg_comp60675_c0	5.1.3.15	945	2220.81	92.07
Glucose-6-phosphate 1-epimerase	K01792	Sg_comp24639_c0	5.1.3.15	237	2	2.09
Glucose-6-phosphate 1-epimerase	K01792	Sg_comp77550_c0	5.1.3.15	1080	851.24	44.26
Glucose-6-phosphate 1-epimerase	K01792	Sg_comp67187_c0	5.1.3.15	831	111.66	8.97
Glucose-6-phosphate 1-epimerase	K01792	Sg_comp71791_c0	5.1.3.15	747	218.03	12.64
Glucose-6-phosphate 1-epimerase	K01792	Sg_comp69144_c0	5.1.3.15	942	262	8.92
Glucose-6-phosphate 1-epimerase	K01792	Sg_comp75228_c2	5.1.3.15	990	725.2	32.28
Glucose-6-phosphate 1-epimerase	K01792	Sg_comp73022_c0	5.1.3.15	123	1661.1	61.86
Glyceraldehyde 3-phosphate dehydrogenase	K00134	Sg_comp58738_c0	1.2.1.12	477	59	11.24
Glyceraldehyde 3-phosphate dehydrogenase	K00134	Sg_comp58738_c1	1.2.1.12	309	70	9.19
Glyceraldehyde 3-phosphate dehydrogenase	K00134	Sg_comp70012_c0	1.2.1.12	1044	39108.56	1312
Glyceraldehyde 3-phosphate dehydrogenase	K00134	Sg_comp72697_c0	1.2.1.12	1068	2466	159.65
Glyceraldehyde 3-phosphate dehydrogenase	K00134	Sg_comp46734_c0	1.2.1.12	273	21	8.76
Glyceraldehyde 3-phosphate dehydrogenase	K00134	Sg_comp40669_c0	1.2.1.12	243	6	1.02
Glyceraldehyde 3-phosphate dehydrogenase	K00134	Sg_comp73685_c0	1.2.1.12	1326	1219.97	39.63
Glyceraldehyde 3-phosphate dehydrogenase	K00134	Sg_comp488329_c0	1.2.1.12	462	10	1.05
Glyceraldehyde 3-phosphate dehydrogenase	K00134	Sg_comp467803_c0	1.2.1.12	210	2	3.93
Glyceraldehyde 3-phosphate dehydrogenase	K00134	Sg_comp13521_c0	1.2.1.12	300	5	2.31
Glyceraldehyde 3-phosphate dehydrogenase	K00134	Sg_comp251097_c0	1.2.1.12	714	22	1.96
Glyceraldehyde 3-phosphate dehydrogenase	K00134	Sg_comp13400_c0	1.2.1.12	288	2	1.06
Phosphoenolpyruvatecarboxykinase (ATP)	K01610	Sg_comp78054_c0	4.1.1.49	1986	6875.56	158.79
Phosphoenolpyruvatecarboxykinase (ATP)	K01610	Sg_comp71567_c0	4.1.1.49	1962	387.76	16.03
Phosphoenolpyruvatecarboxykinase (ATP)	K01610	Sg_comp590003_c0	4.1.1.49	312	2	0.83
Phosphoglycerate kinase	K00927	Sg_comp75541_c0	2.7.2.3	1620	18236.3	512.18
Phosphoglycerate kinase	K00927	Sg_comp73834_c0	2.7.2.3	1296	6196.36	202.6
Alcohol dehydrogenase	K00001	Sg_comp719532_c0	1.1.1.1	219	0	0
Alcohol dehydrogenase	K00001	Sg_comp385573_c0	1.1.1.1	195	4	0.93
Alcohol dehydrogenase	K00001	Sg_comp61119_c0	1.1.1.1	1143	1661.45	92.29
Alcohol dehydrogenase	K00001	Sg_comp72701_c0	1.1.1.1	1143	263	17.81

Gene Name	KEGG Entry	UniGene ID	EC. No.	Gene Length	Reads in Leaf	FPKM
Alcohol dehydrogenase	K00001	Sg_comp53469_c0	1.1.1.1	834	1312.3	45.2
Triosephosphateisomerase (TIM)	K01803	Sg_comp70857_c0	5.3.1.1	1041	7821.87	325.16
Triosephosphateisomerase (TIM)	K01803	Sg_comp73965_c0	5.3.1.1	783	5449.28	266.3
Triosephosphateisomerase (TIM)	K01803	Sg_comp34150_c0	5.3.1.1	219	4	3.71
Pyruvate dehydrogenase E1 component subunit alpha	K00161	Sg_comp72377_c0	1.2.4.1	1500	1933.09	54.8
Pyruvate dehydrogenase E1 component subunit alpha	K00161	Sg_comp58207_c0	1.2.4.1	297	18	8.57
Pyruvate dehydrogenase E1 component subunit alpha	K00161	Sg_comp72419_c0	1.2.4.1	1098	1310.48	126.58
Pyruvate dehydrogenase E1 component subunit alpha	K00161	Sg_comp51629_c0	1.2.4.1	87	9	4.08
Pyruvate dehydrogenase E1 component subunit alpha	K00161	Sg_comp67239_c0	1.2.4.1	612	348	26.75
Pyruvate dehydrogenase E1 component subunit beta	K00162	Sg_comp71989_c0	1.2.4.1	1221	2853.53	108.43
Pyruvate dehydrogenase E1 component subunit beta	K00162	Sg_comp78467_c0	1.2.4.1	1245	633.59	26.21
Pyruvate dehydrogenase E1 component subunit beta	K00162	Sg_comp76290_c0	1.2.4.1	1293	2274.27	84.72
Pyruvate dehydrogenase E1 component subunit beta	K00162	Sg_comp32264_c0	1.2.4.1	375	22	2.68
Pyruvate dehydrogenase E1 component subunit beta	K00162	Sg_comp31023_c0	1.2.4.1	645	29	1.65
Aldehyde dehydrogenase (NAD+)	K00128	Sg_comp60779_c0	1.2.1.3	1119	47	2.13
Aldehyde dehydrogenase (NAD+)	K00128	Sg_comp438039_c0	1.2.1.3	207	2	4.26
Aldehyde dehydrogenase (NAD+)	K00128	Sg_comp162253_c0	1.2.1.3	642	36	2.81
Aldehyde dehydrogenase (NAD+)	K00128	Sg_comp3420_c0	1.2.1.3	321	9	3.42
Aldehyde dehydrogenase (NAD+)	K00128	Sg_comp77091_c0	1.2.1.3	825	1120.34	31.06
Aldehyde dehydrogenase (NAD+)	K00128	Sg_comp768706_c0	1.2.1.3	210	0	0
Aldehyde dehydrogenase (NAD+)	K00128	Sg_comp500774_c0	1.2.1.3	228	2	2.64
Aldehyde dehydrogenase (NAD+)	K00128	Sg_comp238537_c0	1.2.1.3	873	34	2.05
Aldehyde dehydrogenase (NAD+)	K00128	Sg_comp73968_c0	1.2.1.3	1449	1449.47	52.09
Aldehyde dehydrogenase (NAD+)	K00128	Sg_comp76104_c0	1.2.1.3	1635	3444.91	89.6
Aldose 1-epimerase	K01785	Sg_comp63258_c0	5.1.3.3	1140	1407.11	54.55
Aldose 1-epimerase	K01785	Sg_comp77100_c1	5.1.3.3	1077	615	25.83
Aldose 1-epimerase	K01785	Sg_comp68454_c0	5.1.3.3	744	105.93	15.17
Pyruvate decarboxylase	K01568	Sg_comp73692_c0	4.1.1.1	1917	875.92	26.97
Pyruvate decarboxylase	K01568	Sg_comp74559_c0	4.1.1.1	1833	486	10.98
Dihydrolipoamide dehydrogenase	K00382	Sg_comp76842_c0	1.8.1.4	1824	774.36	18.22
Dihydrolipoamide dehydrogenase	K00382	Sg_comp67542_c0	1.8.1.4	1740	940	25.57
Dihydrolipoamide dehydrogenase	K00382	Sg_comp80701_c0	1.8.1.4	1539	4195.15	105
Dihydrolipoamide dehydrogenase	K00382	Sg_comp67118_c0	1.8.1.4	1008	157	7.87
S-(hydroxymethyl)glutathione dehydrogenase / alcohol dehydrogenase	K00121	Sg_comp74135_c0	1.1.1.284, 1.1.1.1	117	2233.47	88.32
S-(hydroxymethyl)glutathione dehydrogenase / alcohol dehydrogenase	K00121	Sg_comp60816_c1	1.1.1.284, 1.1.1.1	138	87	8.74
S-(hydroxymethyl)glutathione dehydrogenase / alcohol dehydrogenase	K00121	Sg_comp60816_c0	1.1.1.284, 1.1.1.1	708	147	14.62
S-(hydroxymethyl)glutathione dehydrogenase / alcohol dehydrogenase	K00121	Sg_comp74850_c0	1.1.1.284, 1.1.1.1	1167	5920.86	236.98
S-(hydroxymethyl)glutathione dehydrogenase / alcohol dehydrogenase	K00121	Sg_comp72558_c0	1.1.1.284, 1.1.1.1	171	2807.68	87.45
Probable phosphoglyceratemutase	K15634	Sg_comp489590_c0	5.4.2.12	234	2	2.13
Probable phosphoglyceratemutase	K15634	Sg_comp68226_c0	5.4.2.12	1602	2050.81	76.4

Gene Name	KEGG Entry	UniGene ID	EC. No.	Gene Length	Reads in Leaf	FPKM
2,3-bisphosphoglycerate-independent phosphoglyceratemutase	K15633	Sg_comp74489_c0	5.4.2.12	1686	2881.36	74.24
2,3-bisphosphoglycerate-independent phosphoglyceratemutase	K15633	Sg_comp287241_c0	5.4.2.12	288	2	1.03
2,3-bisphosphoglycerate-independent phosphoglyceratemutase	K15633	Sg_comp32332_c0	5.4.2.12	1161	55	2.43
