# Supplementary material for: Harnessing de novo transcriptome sequencing to identify and characterize genes regulating carbohydrate biosynthesis pathways in Salvia guaranitica L
Source: Front Plant Sci. 2024 Sep 26;15:1467432. doi: 10.3389/fpls.2024.1467432 (PMC11464306; doi:10.3389/fpls.2024.1467432)
Supplement: Supplementary file 5 [file Table5.pdf]

Supplementary Material

Table S5. Transcript Levels of the genes regulating starch and sucrose biosynthesis as per their annotations.

Pathway / *Starch & Sucrose Metabolism*						
Gene Name	**KEGG Entry**	**UniGene ID**	**EC. No.**	**Gene Length**	**Reads in Leaf**	**FPKM**
Starch synthase (SS1)	K00703	Sg_comp78845_c1	2.4.1.21	267	20	14.37
Starch synthase	K00703	Sg_comp76497_c0	2.4.1.21	759	792.07	36.25
Starch synthase	K00703	Sg_comp43828_c0	2.4.1.21	318	0	0
Starch synthase	K00703	Sg_comp50503_c0	2.4.1.21	357	15	4.54
Starch synthase	K00703	Sg_comp78845_c0	2.4.1.21	2139	4307.97	89.81
Starch synthase	K00703	Sg_comp73757_c0	2.4.1.21	972	3830.37	70
Glucose-1-phosphate adenylyltransferase	K00975	Sg_comp74948_c0	2.7.7.27	1635	7612.34	165.09
Glucose-1-phosphate adenylyltransferase	K00975	Sg_comp13430_c0	2.7.7.27	312	2	0.82
Glucose-1-phosphate adenylyltransferase	K00975	Sg_comp75045_c0	2.7.7.27	1611	944.63	21.02
Glucose-1-phosphate adenylyltransferase	K00975	Sg_comp73024_c0	2.7.7.27	996	4167.77	205.39
Glucose-1-phosphate adenylyltransferase	K00975	Sg_comp74192_c0	2.7.7.27	1344	3534.29	103.46
Glucose-1-phosphate adenylyltransferase	K00975	Sg_comp68939_c0	2.7.7.27	687	2100.86	218.87
Glucose-1-phosphate adenylyltransferase	K00975	Sg_comp75400_c0	2.7.7.27	1563	3008.41	79.94
Glucose-1-phosphate adenylyltransferase	K00975	Sg_comp40161_c0	2.7.7.27	318	4	1.6
Beta-amylase	K01177	Sg_comp70736_c0	3.2.1.2	1680	4307.74	144.95
Beta-amylase	K01177	Sg_comp78265_c0	3.2.1.2	792	1624.01	32.6
Beta-amylase	K01177	Sg_comp73249_c0	3.2.1.2	1677	2279.26	56.47
Beta-amylase	K01177	Sg_comp73960_c0	3.2.1.2	1719	5727.95	140.44
Beta-amylase	K01177	Sg_comp52758_c0	3.2.1.2	438	302	58.67
Endoglucanase	K01179	Sg_comp74963_c0	3.2.1.4	1512	343	12.5
Trehalose 6-phosphate synthase/phosphatase	K16055	Sg_comp26963_c0	2.4.1.15, 3.1.3.12	141	20	8.57
Trehalose 6-phosphate synthase/phosphatase	K16055	Sg_comp80006_c0	2.4.1.15 3,.1.3.12	1596	1265.17	20.02
Trehalose 6-phosphate synthase/phosphatase	K16055	Sg_comp76798_c0	2.4.1.15 3,.1.3.12	1683	379	10.28
Trehalose 6-phosphate synthase/phosphatase	K16055	Sg_comp75039_c0	2.4.1.15 3,.1.3.12	996	249.01	8.94
Trehalose 6-phosphate synthase/phosphatase	K16055	Sg_comp54364_c0	2.4.1.15 3,.1.3.12	255	32	25.38
Trehalose 6-phosphate synthase/phosphatase	K16055	Sg_comp65076_c0	2.4.1.15, 3.1.3.12	1947	104	2.49
Trehalose 6-phosphate synthase/phosphatase	K16055	Sg_comp79940_c0	2.4.1.15 3,.1.3.12	2610	2197.9	35
Trehalose 6-phosphate synthase/phosphatase	K16055	Sg_comp272859_c0	2.4.1.15 3,.1.3.12	273	4	2.45

Gene Name	KEGG Entry	UniGene ID	EC. No.	Gene Length	Reads in Leaf	FPKM
Trehalose 6-phosphate synthase/phosphatase	K16055	Sg_comp56036_c0	2.4.1.15 3,.1.3.12	318	14	2.16
Trehalose 6-phosphate synthase/phosphatase	K16055	Sg_comp52146_c0	2.4.1.15 3,.1.3.12	243	31	30.26
Trehalose 6-phosphate synthase/phosphatase	K16055	Sg_comp80574_c0	2.4.1.15, 3.1.3.12	1365	2781.96	122.55
Trehalose 6-phosphate synthase/phosphatase	K16055	Sg_comp79765_c0	2.4.1.15 3,.1.3.12	2637	2067.05	27.06
Trehalose 6-phosphate synthase/phosphatase	K16055	Sg_comp36814_c0	2.4.1.15 3,.1.3.12	273	2	1.23
Trehalose 6-phosphate synthase/phosphatase	K16055	Sg_comp76640_c0	2.4.1.15 3,.1.3.12	1800	235.3	13.86
Trehalose 6-phosphate synthase/phosphatase	K16055	Sg_comp57125_c0	2.4.1.15 3,.1.3.12	660	18	1.69
Trehalose 6-phosphate synthase/phosphatase	K16055	Sg_comp80221_c0	2.4.1.15 3,.1.3.12	2721	1041.49	15.9
1,4-alpha-glucan branching enzyme	K00700	Sg_comp41027_c0	2.4.1.18	150	3	5.33
1,4-alpha-glucan branching enzyme	K00700	Sg_comp448265_c0	2.4.1.18	261	2	1.42
1,4-alpha-glucan branching enzyme	K00700	Sg_comp62514_c0	2.4.1.18	2442	949	17.59
1,4-alpha-glucan branching enzyme	K00700	Sg_comp80024_c2	2.4.1.18	2637	4235.03	84.58
Glucose-6-phosphate isomerase	K01810	Sg_comp78491_c0	5.3.1.9	1827	2156.03	62.11
Glucose-6-phosphate isomerase	K01810	Sg_comp74788_c0	5.3.1.9	1833	3448.56	77.2
Alpha-amylase	K01176	Sg_comp24129_c0	3.2.1.1	237	4	4.11
Alpha-amylase	K01176	Sg_comp220893_c0	3.2.1.1	267	3	2.04
Alpha-amylase	K01176	Sg_comp70991_c0	3.2.1.1	1197	427	20.41
Alpha-glucosidase	K01187	Sg_comp74598_c0	3.2.1.20	3072	5261.47	73.3
Alpha-glucosidase	K01187	Sg_comp77637_c0	3.2.1.20	1830	2370	39.94
Alpha-glucosidase	K01187	Sg_comp79493_c0	3.2.1.20	1194	1259.99	76.71
Alpha-glucosidase	K01187	Sg_comp483158_c0	3.2.1.20	210	7	1.71
Phosphoglucomutase	K01835	Sg_comp37833_c0	5.4.2.2	291	7	3.62
Phosphoglucomutase	K01835	Sg_comp78938_c0	5.4.2.2	1953	6893.07	214.26
Phosphoglucomutase	K01835	Sg_comp514846_c0	5.4.2.2	219	1	1.5
Phosphoglucomutase	K01835	Sg_comp44304_c0	5.4.2.2	207	22	46.89
Phosphoglucomutase	K01835	Sg_comp376934_c0	5.4.2.2	225	1	1.35
Phosphoglucomutase	K01835	Sg_comp79118_c0	5.4.2.2	1758	2920.12	255.54
Phosphoglucomutase	K01835	Sg_comp37833_c1	5.4.2.2	300	2	0.91
Beta-glucosidase	K01188	Sg_comp45303_c0	3.2.1.21	246	8	7.42
Beta-glucosidase	K01188	Sg_comp74724_c0	3.2.1.21	210	2569.03	77.34
Beta-glucosidase	K01188	Sg_comp47619_c0	3.2.1.21	573	20	5.6
Beta-glucosidase	K01188	Sg_comp485487_c0	3.2.1.21	204	0	0
Beta-glucosidase	K01188	Sg_comp374340_c0	3.2.1.21	366	5	1.39
Beta-glucosidase	K01188	Sg_comp54969_c0	3.2.1.21	1554	74	2.24
Hexokinase	K00844	Sg_comp77418_c0	2.7.1.1	1587	174	4.32
Hexokinase	K00844	Sg_comp37299_c0	2.7.1.1	426	6	1.22
Hexokinase	K00844	Sg_comp385086_c0	2.7.1.1	222	2	2.49
Hexokinase	K00844	Sg_comp4335_c0	2.7.1.1	300	5	2.36
Hexokinase	K00844	Sg_comp73293_c0	2.7.1.1	258	665.14	35.2
Hexokinase	K00844	Sg_comp77187_c0	2.7.1.1	1536	2010.24	49.58
Hexokinase	K00844	Sg_comp57244_c0	2.7.1.1	273	23	4.11
Hexokinase	K00844	Sg_comp74205_c0	2.7.1.1	1266	146.47	8.63
4-alpha-glucanotransferase	K00705	Sg_comp75188_c0	2.4.1.25	1731	751.42	20.38
4-alpha-glucanotransferase	K00705	Sg_comp79639_c0	2.4.1.25	2922	2050.12	31.04

Gene Name	KEGG Entry	UniGene ID	EC. No.	Gene Length	Reads in Leaf	FPKM
Starch phosphorylase	K00688	Sg_comp80005_c0	2.4.1.1	2553	3188.24	50.29
Starch phosphorylase	K00688	Sg_comp63078_c0	2.4.1.1	299	7	4.46
Starch phosphorylase	K00688	Sg_comp175535_c0	2.4.1.1	108	0	0
Starch phosphorylase	K00688	Sg_comp50855_c0	2.4.1.1	117	17	10.18
Starch phosphorylase	K00688	Sg_comp79295_c0	2.4.1.1	2979	676	14.84
Starch phosphorylase	K00688	Sg_comp78939_c0	2.4.1.1	2694	3659.54	56.6
Starch phosphorylase	K00688	Sg_comp79722_c0	2.4.1.1	789	1539.45	28.18
Starch phosphorylase	K00688	Sg_comp59932_c0	2.4.1.1	372	47	12.71
UDPglucose 6-dehydrogenase	K00012	Sg_comp51715_c0	1.1.1.22	603	32	4.46
UDPglucose 6-dehydrogenase	K00012	Sg_comp70280_c0	1.1.1.22	933	540	38.27
UDPglucose 6-dehydrogenase	K00012	Sg_comp70280_c1	1.1.1.22	543	338	37.18
UDPglucose 6-dehydrogenase	K00012	Sg_comp75333_c0	1.1.1.22	1536	1915	51.01
UDPglucose 6-dehydrogenase	K00012	Sg_comp58317_c0	1.1.1.22	855	36	2.26
UDPglucose 6-dehydrogenase	K00012	Sg_comp235962_c0	1.1.1.22	141	4	0.87
Glycogen operon protein	K02438	Sg_comp78888_c0	3.2.1.-	534	955.01	27.42
Glycogen operon protein	K02438	Sg_comp78253_c0	3.2.1.-	1317	943.77	42.36
Trehalose 6-phosphate phosphatase	K01087	Sg_comp61205_c0	3.1.3.12	1041	65	2.5
Trehalose 6-phosphate phosphatase	K01087	Sg_comp72173_c0	3.1.3.12	903	94	9.07
Trehalose 6-phosphate phosphatase	K01087	Sg_comp72011_c0	3.1.3.12	1158	534.01	26.13
Trehalose 6-phosphate phosphatase	K01087	Sg_comp54278_c0	3.1.3.12	195	13	2.83
Trehalose 6-phosphate phosphatase	K01087	Sg_comp76143_c0	3.1.3.12	1146	538.02	15.4
Trehalose 6-phosphate phosphatase	K01087	Sg_comp8100_c0	3.1.3.12	300	4	1.85
Trehalose 6-phosphate phosphatase	K01087	Sg_comp34086_c0	3.1.3.12	489	14	1.46
Fructokinase	K00847	Sg_comp71340_c1	2.7.1.4	1137	5659.29	274.55
Fructokinase	K00847	Sg_comp60127_c0	2.7.1.4	393	44	6.43
Fructokinase	K00847	Sg_comp6777_c0	2.7.1.4	693	13	1.17
Fructokinase	K00847	Sg_comp75813_c0	2.7.1.4	1143	1179.48	34.68
Beta-D-xylosidase 4	K15920	Sg_comp55191_c0	3.2.1.37	294	63.87	12.88
Beta-D-xylosidase 4	K15920	Sg_comp78566_c0	3.2.1.37	2340	695.99	17.8
UTP--glucose-1-phosphate uridylyltransferase	K00963	Sg_comp78980_c0	2.7.7.9	1437	3033.7	100.06
UTP--glucose-1-phosphate uridylyltransferase	K00963	Sg_comp752238_c0	2.7.7.9	249	1	0.88
1,4-beta-D-xylan synthase	K00770	Sg_comp79298_c0	2.4.2.24	3378	392.01	9.77
Beta-glucosidase	K05349	Sg_comp71492_c0	3.2.1.21	147	920.01	29.27
Beta-glucosidase	K05349	Sg_comp78641_c0	3.2.1.21	1113	5917.45	119.73
Beta-glucosidase	K05349	Sg_comp72868_c0	3.2.1.21	618	446	70.08
Beta-glucosidase	K05349	Sg_comp49871_c0	3.2.1.21	231	2	1.01
Beta-glucosidase	K05349	Sg_comp317171_c0	3.2.1.21	462	6	1.06
Beta-glucosidase	K05349	Sg_comp618239_c0	3.2.1.21	204	1	2.13
Beta-glucosidase	K05349	Sg_comp71492_c1	3.2.1.21	291	150.55	29.03
Beta-glucosidase	K05349	Sg_comp50418_c0	3.2.1.21	336	6	1.37
Beta-glucosidase	K05349	Sg_comp77885_c0	3.2.1.21	1074	329.24	7.79
Beta-glucosidase	K05349	Sg_comp59184_c0	3.2.1.21	903	34	3.01
Beta-glucosidase	K05349	Sg_comp76336_c0	3.2.1.21	1662	515.01	13.15
Beta-glucosidase	K05349	Sg_comp278756_c0	3.2.1.21	210	3	0.83
Beta-glucosidase	K05349	Sg_comp621348_c0	3.2.1.21	129	0	0
Beta-glucosidase	K05349	Sg_comp427999_c0	3.2.1.21	243	2	1.48

Gene Name	KEGG Entry	UniGene ID	EC. No.	Gene Length	Reads in Leaf	FPKM
Beta-glucosidase	K05349	Sg_comp423407_c0	3.2.1.21	354	14	4.18
Beta-glucosidase	K05349	Sg_comp76294_c0	3.2.1.21	2292	698	14.45
Beta-glucosidase	K05349	Sg_comp64719_c0	3.2.1.21	816	32.56	2.42
Beta-glucosidase	K05349	Sg_comp23791_c0	3.2.1.21	405	13	2.04
Beta-glucosidase	K05349	Sg_comp76779_c0	3.2.1.21	1962	409	10.7
Beta-glucosidase	K05349	Sg_comp29730_c0	3.2.1.21	183	1	0.54
Beta-glucosidase	K05349	Sg_comp72868_c1	3.2.1.21	1260	1005.89	43.2
Beta-glucosidase	K05349	Sg_comp58296_c0	3.2.1.21	231	14	2.28
Beta-fructofuranosidase	K01193	Sg_comp76634_c0	3.2.1.26	1614	5381.11	121.23
Beta-fructofuranosidase	K01193	Sg_comp58649_c0	3.2.1.26	387	75	18.73
Beta-fructofuranosidase	K01193	Sg_comp56943_c0	3.2.1.26	135	14	9.15
Beta-fructofuranosidase	K01193	Sg_comp75917_c0	3.2.1.26	1521	1820.77	55.9
Beta-fructofuranosidase	K01193	Sg_comp437927_c0	3.2.1.26	186	1	1.92
Beta-fructofuranosidase	K01193	Sg_comp72213_c0	3.2.1.26	375	292.56	8.19
Alpha-1,4-galacturonosyltransferase	K13648	Sg_comp492104_c0	2.4.1.43	255	2	1.63
Alpha-1,4-galacturonosyltransferase	K13648	Sg_comp75346_c0	2.4.1.43	2097	458.74	10.76
Alpha-1,4-galacturonosyltransferase	K13648	Sg_comp79040_c0	2.4.1.43	1908	628.9	13.16
Alpha-1,4-galacturonosyltransferase	K13648	Sg_comp13649_c0	2.4.1.43	243	0	0
Alpha-1,4-galacturonosyltransferase	K13648	Sg_comp66201_c0	2.4.1.43	498	142	22.04
Alpha-1,4-galacturonosyltransferase	K13648	Sg_comp62617_c0	2.4.1.43	348	37	2.83
Alpha-1,4-galacturonosyltransferase	K13648	Sg_comp50298_c0	2.4.1.43	465	11	1.91
Alpha-1,4-galacturonosyltransferase	K13648	Sg_comp79851_c0	2.4.1.43	1992	1512.35	31.03
Alpha-1,4-galacturonosyltransferase	K13648	Sg_comp79897_c0	2.4.1.43	1689	875.65	52.38
Alpha-1,4-galacturonosyltransferase	K13648	Sg_comp74594_c0	2.4.1.43	1743	2310.93	59.69
Alpha-1,4-galacturonosyltransferase	K13648	Sg_comp59595_c0	2.4.1.43	483	31	5.03
Alpha-1,4-galacturonosyltransferase	K13648	Sg_comp60156_c0	2.4.1.43	279	12	6.86
Alpha-1,4-galacturonosyltransferase	K13648	Sg_comp77582_c0	2.4.1.43	1869	997.1	21.88
Alpha-1,4-galacturonosyltransferase	K13648	Sg_comp78700_c0	2.4.1.43	1929	667.94	16.99
Alpha-1,4-galacturonosyltransferase	K13648	Sg_comp34593_c0	2.4.1.43	216	3	5.07
Alpha-1,4-galacturonosyltransferase	K13648	Sg_comp75200_c2	2.4.1.43	1701	946	27.88
Alpha-1,4-galacturonosyltransferase	K13648	Sg_comp77988_c0	2.4.1.43	1644	246	9.25
Alpha-1,4-galacturonosyltransferase	K13648	Sg_comp80102_c0	2.4.1.43	2055	1660.08	38.07
Alpha-1,4-galacturonosyltransferase	K13648	Sg_comp78859_c0	2.4.1.43	309	1241.33	24.35
Alpha-1,4-galacturonosyltransferase	K13648	Sg_comp60068_c0	2.4.1.43	102	30	16.19
Alpha-1,4-galacturonosyltransferase	K13648	Sg_comp76415_c0	2.4.1.43	1851	852.17	19.12

Gene Name	KEGG Entry	UniGene ID	EC. No.	Gene Length	Reads in Leaf	FPKM
Alpha-1,4-galacturonosyltransferase	K13648	Sg_comp77441_c0	2.4.1.43	1326	538.03	12.99
Alpha,alpha-trehalase	K01194	Sg_comp61483_c1	3.2.1.28	834	28	2.01
Alpha,alpha-trehalase	K01194	Sg_comp41541_c0	3.2.1.28	393	11	2.63
Alpha,alpha-trehalase	K01194	Sg_comp61483_c0	3.2.1.28	594	13	1.47
Sucrase-isomaltase / oligo-1,6-glucosidase	K01203	Sg_comp794118_c0	3.2.1.48, 3.2.1.10	234	1	1.15
Pectinesterase	K01051	Sg_comp199827_c0	3.1.1.11	291	11	5.87
Pectinesterase	K01051	Sg_comp67061_c0	3.1.1.11	279	3	1.75
Pectinesterase	K01051	Sg_comp31227_c0	3.1.1.11	588	21	2.47
Pectinesterase	K01051	Sg_comp71329_c0	3.1.1.11	846	316.16	21.26
Pectinesterase	K01051	Sg_comp54135_c0	3.1.1.11	222	0	0
Pectinesterase	K01051	Sg_comp427469_c0	3.1.1.11	198	1	2.61
Pectinesterase	K01051	Sg_comp61829_c0	3.1.1.11	558	103	9.17
Pectinesterase	K01051	Sg_comp55583_c0	3.1.1.11	330	13	4.6
Pectinesterase	K01051	Sg_comp67061_c1	3.1.1.11	543	759.2	132.62
Pectinesterase	K01051	Sg_comp71238_c0	3.1.1.11	1221	171	6.84
Pectinesterase	K01051	Sg_comp72897_c0	3.1.1.11	1239	690.21	35.9
Pectinesterase	K01051	Sg_comp593880_c0	3.1.1.11	258	2	0.85
Pectinesterase	K01051	Sg_comp75132_c0	3.1.1.11	1071	326.98	13.08
Pectinesterase	K01051	Sg_comp65752_c0	3.1.1.11	729	41	3.04
Pectinesterase	K01051	Sg_comp59935_c0	3.1.1.11	501	31	4.04
Pectinesterase	K01051	Sg_comp53147_c0	3.1.1.11	930	37	2.11
Pectinesterase	K01051	Sg_comp71257_c0	3.1.1.11	924	143.99	11.96
Pectinesterase	K01051	Sg_comp29520_c0	3.1.1.11	348	8	2.85
Pectinesterase	K01051	Sg_comp47590_c0	3.1.1.11	342	8	2.5
Pectinesterase	K01051	Sg_comp75144_c0	3.1.1.11	1806	27010	898.23
Pectinesterase	K01051	Sg_comp9366_c0	3.1.1.11	264	7	2.25
Pectinesterase	K01051	Sg_comp36000_c0	3.1.1.11	369	13	3.54
Pectinesterase	K01051	Sg_comp70209_c0	3.1.1.11	993	611	33.78
Pectinesterase	K01051	Sg_comp1013565_c0	3.1.1.11	276	1	0.61
Pectinesterase	K01051	Sg_comp75144_c1	3.1.1.11	534	10639.64	1518.69
Glucuronosyltransferase	K00699	Sg_comp755975_c0	2.4.1.17	204	0	0
UDP-glucuronate 4-epimerase	K08679	Sg_comp73047_c0	5.1.3.6	600	287.22	18.19
UDP-glucuronate 4-epimerase	K08679	Sg_comp38928_c0	5.1.3.6	456	58.99	5.14
UDP-glucuronate 4-epimerase	K08679	Sg_comp74731_c1	5.1.3.6	1305	3587.69	219.56
UDP-glucuronate 4-epimerase	K08679	Sg_comp71078_c1	5.1.3.6	177	355.87	70.22
UDP-glucuronate 4-epimerase	K08679	Sg_comp70949_c1	5.1.3.6	210	7	13.77
UDP-glucuronate 4-epimerase	K08679	Sg_comp76845_c0	5.1.3.6	1368	1519.61	25.85
UDP-glucuronate 4-epimerase	K08679	Sg_comp56210_c0	5.1.3.6	198	81.5	19.92
UDP-glucuronate 4-epimerase	K08679	Sg_comp70949_c0	5.1.3.6	585	219.9	24.85
UDP-glucuronate 4-epimerase	K08679	Sg_comp58919_c0	5.1.3.6	231	182.95	20.42
UDP-glucuronate 4-epimerase	K08679	Sg_comp38928_c1	5.1.3.6	867	57	3.9
UDP-glucuronate 4-epimerase	K08679	Sg_comp74197_c0	5.1.3.6	1803	650.75	24.76
UDP-glucuronate 4-epimerase	K08679	Sg_comp71078_c0	5.1.3.6	876	719	52.67
UDP-glucuronate 4-epimerase	K08679	Sg_comp424779_c0	5.1.3.6	288	3	1.08
Lysosomal alpha-glucosidase	K12316	Sg_comp769274_c0	3.2.1.20	228	0	0
Lysosomal alpha-glucosidase	K12316	Sg_comp769134_c0	3.2.1.20	204	1	2.32
Sucrose-phosphate synthase	K00696	Sg_comp75149_c0	2.4.1.14	327	1098	15.71
Sucrose-phosphate synthase	K00696	Sg_comp79881_c0	2.4.1.14	3132	2274.47	33.58
Sucrose-phosphate synthase	K00696	Sg_comp57107_c0	2.4.1.14	939	27	1.67
Sucrose-phosphate synthase	K00696	Sg_comp67830_c0	2.4.1.14	1857	78	2.11
Sucrose-phosphate synthase	K00696	Sg_comp48182_c0	2.4.1.14	318	9	1.18
Sucrose-phosphate synthase	K00696	Sg_comp210737_c0	2.4.1.14	381	12	2.28
Sucrose-phosphate synthase	K00696	Sg_comp72520_c0	2.4.1.14	1422	249.52	7.27

Gene Name	KEGG Entry	UniGene ID	EC. No.	Gene Length	Reads in Leaf	FPKM
Sucrose-phosphate synthase	K00696	Sg_comp52682_c0	2.4.1.14	246	5	4.64
Sucrose-phosphate synthase	K00696	Sg_comp64610_c0	2.4.1.14	1104	44	2.22
Sucrose-phosphate synthase	K00696	Sg_comp32614_c0	2.4.1.14	720	29	2.55
Sucrose-phosphate synthase	K00696	Sg_comp67495_c0	2.4.1.14	939	119	10.59
Sucrose synthase	K00695	Sg_comp38545_c0	2.4.1.13	225	2	2.76
Sucrose synthase	K00695	Sg_comp77396_c0	2.4.1.13	2517	213	3.94
Sucrose synthase	K00695	Sg_comp78109_c0	2.4.1.13	2445	3159.98	51.47
Sucrose synthase	K00695	Sg_comp68172_c0	2.4.1.13	360	99.97	3.18
Sucrose synthase	K00695	Sg_comp77363_c0	2.4.1.13	2439	5651.52	138.51
Sucrose synthase	K00695	Sg_comp66020_c0	2.4.1.13	516	75	9.18
