# Supplementary material for: Harnessing de novo transcriptome sequencing to identify and characterize genes regulating carbohydrate biosynthesis pathways in Salvia guaranitica L
Source: Front Plant Sci. 2024 Sep 26;15:1467432. doi: 10.3389/fpls.2024.1467432 (PMC11464306; doi:10.3389/fpls.2024.1467432)
Supplement: Supplementary file 6 [file Table6.pdf]

Supplementary Material

Table S6. Sage genes and primers utilized for qRT-PCR and Semi-qRT-PCR.

UniGene ID	Primer Name	Primer Sequence	PCR product (bp)
**SgB-ACTIN**	Beta-actin -F	5′- CTGGATTTGCGGGAGATG -3′	166
	Beta-actin -R	5′- CCGTGCTCAATTGGATACTT -3′	
**SgGPI**	Glucose-6-phosphate isomerase-F	5′- AGTGGTTTCAAAGAGTGGAG -3′	151
	Glucose-6-phosphate isomerase-R	5′- CTCAATCCTGGCTGTGTTAT-3′	
**SgT6PS**	Trehalose 6-phosphate synthase/phosphatase-F	5′- CTCTGACTGCTGTTCCTATTC-3′	148
	Trehalose 6-phosphate synthase/phosphatase-R	5′- TCTTGACATGCCACAAACT-3′	
**SgSUS**	Sucrose synthase-F	5′- TCGACCACCATGGAGAAA -3′	150
	Sucrose synthase-R	5′- CCCACTCTTTGAACCTCATC -3′	
**SgPFK9**	6-phosphofructokinase 1-F	5′- GAATTCAGGTGAAGGCAGAG -3′	157
	6-phosphofructokinase 1-R	5′- CAGAAGGTGGAAGGATAGGA-3′	
**SgALDH**	Aldehyde dehydrogenase family 7 member A1-F	5′- AGTTCAAAGGTGGGTCTAATG -3′	149
	Aldehyde dehydrogenase family 7 member A1-R	5′- CCAACAGCAGCGAATAGAA -3′	
**SgALDO**	Fructose-bisphosphate aldolase, class I-F	5′- CGGGATCAAAGTCGACAAG -3′	142
	Fructose-bisphosphate aldolase, class I-R	5′- CACAACGGTCCTCCATTTAG -3′	
**SgPYK**	Pyruvate kinase-F	5′- ACAGAGAAGTCTCCGAATCA -3′	157
	Pyruvate kinase-R	5′- AGGAACACTGACCACAAATC -3′	
**SgFBP**	Fructose-1,6-bisphosphatase I-F	5′- TCTCTCCAGAGTGGAACTAAG -3′	148
	Fructose-1,6-bisphosphatase I-R	5′- TAATGTCTAGGTGGGTCAGG -3′	
**SgACS**	Acetyl-CoA synthetase-F	5′- CTGCCTTGGAGAAGGAAAG -3′	145
	Acetyl-CoA synthetase-R	5′- GTGCTCCCACTGGTATAAAG -3′	
**SgPCKA**	Phosphoenolpyruvate carboxykinase (ATP)-F	5′- GCAACCGATACACTCACTAC -3′	146
	Phosphoenolpyruvate carboxykinase (ATP)-R	5′- GCGCTTAGGCATGAGATAG -3′	
**SgGlGA**	Starch synthase-F	5′- TCTGGAAAGGTTGAGTGTAAG -3′	157
	Starch synthase-R	5′- GGACATCATCGTGCATAAGA -3′	
**SgGlGC**	Glucose-1-phosphate adenylyltransferase -F	5′- CTCGAACATATACTGGGAATGG -3′	154
	Glucose-1-phosphate adenylyltransferase -R	5′- GCCTTGGCATCCTCAAATA -3′	
**SgBMY**	Beta-amylase-F	5′- GCCGGAAGATACCAATTTCT-3′	149
	Beta-amylase-R	5′- CACCCTTGTTCTCGAATATGT-3′	
**SgGBE1**	1,4-alpha-glucan branching enzyme-F	5′- GCCATACCACACAACTCAA -3′	140
	1,4-alpha-glucan branching enzyme-R	5′- TCCCAGTACACACCATCATA -3′	
**Sg AGL**	Alpha-glucosidase-F	5′- TTCCAAATGGAGAGGCAATAG -3′	150
	Alpha-glucosidase-R	5′- CAAGGACATCAGTTGGTGAA -3′	
**SgBGL**	Beta-glucosidase-F	5′- GGAATGGACGATGGAAACA -3′	151
	Beta-glucosidase-R	5′- GCATCGACCAAACGAAGTA -3′	
**SgHK**	Hexokinase-F	5′- GGTAGAGGAAACACGAGAAC -3′	155
	Hexokinase-R	5′- GAGACATTGCCTCCTGTAAG -3′	
**SgPYG**	Starch phosphorylase-F	5′- CTGAGGAGAGGAGAGAAAGA-3′	153
	Starch phosphorylase-R	5′- CCTTTAGAAGGTCGCCTATG -3′	
**SgUGDH**	UDPglucose 6-dehydrogenase-F	5′- ACTCAAGTGCCCTGATATTG-3′	155
	UDPglucose 6-dehydrogenase-R	5′- CCACATCTGTGCTGAAGAA-3′	
**SgINV**	Beta-fructofuranosidase-F	5′- CGCATCACGATTGGTTCT-3′	157
	Beta-fructofuranosidase-R	5′- CGGTCAAAGAAACTGGGTAA-3′	
**At-B-actin**	Beta-actin -F	5′- GGCTGAGGCTGATGATATTC-3′	155
	Beta-actin -R	5′- CCTTCTGGTTCATCCCAAC-3′	
